# Neural Correlates of Natural Human Echolocation in Early and Late Blind Echolocation Experts

**DOI:** 10.1371/journal.pone.0020162

**Published:** 2011-05-25

**Authors:** Lore Thaler, Stephen R. Arnott, Melvyn A. Goodale

**Affiliations:** 1 Department of Psychology, University of Western Ontario, London, Ontario, Canada; 2 Rotman Research Institute, Baycrest, Toronto, Ontario, Canada; Istituto di Neuroscienze, Italy

## Abstract

**Background:**

A small number of blind people are adept at echolocating silent objects simply by producing mouth clicks and listening to the returning echoes. Yet the neural architecture underlying this type of aid-free human echolocation has not been investigated. To tackle this question, we recruited echolocation experts, one early- and one late-blind, and measured functional brain activity in each of them while they listened to their own echolocation sounds.

**Results:**

When we compared brain activity for sounds that contained both clicks and the returning echoes with brain activity for control sounds that did not contain the echoes, but were otherwise acoustically matched, we found activity in calcarine cortex in both individuals. Importantly, for the same comparison, we did not observe a difference in activity in auditory cortex. In the early-blind, but not the late-blind participant, we also found that the calcarine activity was greater for echoes reflected from surfaces located in contralateral space. Finally, in both individuals, we found activation in middle temporal and nearby cortical regions when they listened to echoes reflected from moving targets.

**Conclusions:**

These findings suggest that processing of click-echoes recruits brain regions typically devoted to vision rather than audition in both early and late blind echolocation experts.

## Introduction

**Figure 1:**
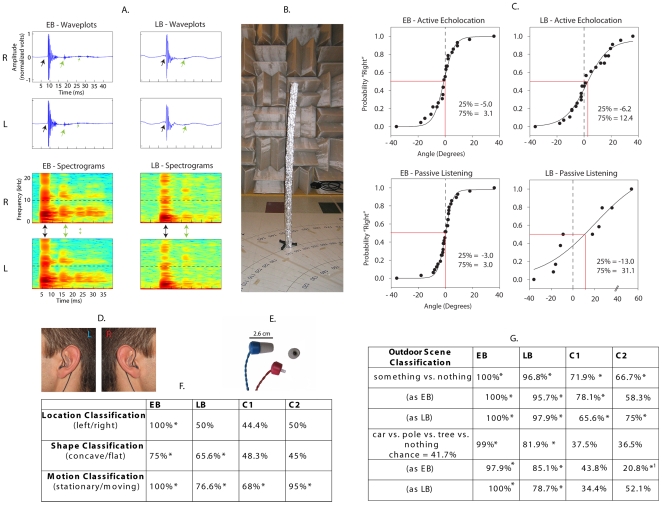
Illustration of click sounds, click echoes and experimental materials, and summary of behavioural results. **A:** Waveplots and spectrograms of the sound of a click (highlighted with black arrows) and its echo (highlighted with green arrows) recorded in the left (L) and right (R) ears of EB and LB (sampling rate 44.1 kHz) (Sound S1 and Sound S2). Both EB and LB made the clicks in the presence of a position marker (shown in 1B) located straight ahead. Spectrograms were obtained using an FFT window of 256 samples, corresponding to approximately 5.6 ms in our recordings. Waveform plots and spectrograms are for illustration. While the exact properties of the click and its echo (e.g. loudness, timbre) are specific to the person generating the click as well as the sound reflecting surface, prominent characteristics of clicks are short duration (approximately 10 ms) and broad frequency spectra, both of which are evident in the plots. **B:** Position marker used for angular position discrimination experiments during active echolocation, and to make recordings for the passive listening paradigm. The marker was an aluminium foil covered foam half-tube (diameter 6 cm, height 180 cm), placed vertically, at a distance of 150 cm, with the concave side facing the subject. Note the 125-Hz cutoff wedge system on the walls of the anechoic chamber. **C:** Results of angular position discrimination experiments (for examples of sound stimuli used during passive listening listen to Sounds S5 and S6). Plotted on the ordinate is the probability that the participant judges the position marker to be located to the right of its straight ahead reference position. Plotted on the abscissa is the position of the test position with respect to the straight ahead in degrees. Negative numbers indicate a position shift in the counter clockwise direction. Psychometric functions were obtained by fitting a 3-parameter sigmoid to the data. 25% and 75% thresholds and bias (denoted in red) were estimated from fitted curves. The zero-bias line (dashed line) is drawn for comparison. **D:** Stimuli were recorded with microphones placed in the echolocator's ears, directly in front of the ear canal. **E:** During passive listening, stimuli were delivered using fMRI compatible in-ear headphones, which imposed a 10 kHz cutoff (marked with a dashed line in spectrograms in A). **F–G:** Behavioral results from the various passive-listening classification tasks (for examples of sound stimuli used during the various classification tasks listen to Sound S7, Sound S8, Sound S9, Sound S10, Sound S11, Sound S12, Sound S13). Shown is percentage correct. Asterisks indicate that performance is significantly different from chance (p<.05). Unless otherwise indicated, chance performance is 50%. Sample sizes (reported in Table S1 and Table S2) fulfil minimum requirement for confidence intervals for a proportion based on the normal approximation [48]. ^1^ = *less* than chance, because of bias to classify as ‘tree’.

Research has shown that people, like many animals, are capable of using reflected sound waves (i.e. echoes) to perceive attributes of their silent physical environment (for reviews see [1]–[3]). Although this ability can been promoted through technological aids (e.g. [4]–[7]), such devices are by no means a necessary requirement. Indeed, there is increasing recognition of the fact that some people can actively echolocate without the use of any peripheral aids whatsoever [3]. The enormous potential of this ‘natural’ echolocation ability is realized in a segment of the blind population that has learned to sense silent objects in the environment simply by generating clicks with their tongues and mouths and then listening to the returning echoes [8]. The echolocation click produced by such individuals tends to be short (approximately 10 ms) and spectrally broad (Figure 1A; Sound S1 and Sound S2). Clicks can be produced in various ways, but it has been suggested that the palatal click, produced by quickly moving the tongue backwards and downwards from the palatal region directly behind the teeth, is best for natural human echolocation [9]. For the skilled echolocator, the returning echoes can potentially provide a great deal of information regarding the position, distance, size, shape and texture of objects [3].

To this point, research into natural human echolocation has been exclusively behavioural in nature. As a consequence, the neural processes underlying this ability are completely unknown. Some expectations about these mechanisms can be gathered from a positron emission tomography (PET) study [10] in which participants were trained to localize objects based on auditory input from a sensory substitution device (SSD) that emitted ultrasonic sounds and then transformed any echo information into audible pitch and interaural level differences associated with an object's distance and angular position, respectively [4]. Relative to simple auditory orienting movements of the head toward external noisebursts, early blind subjects, but not sighted controls, showed increased activity in anatomically defined Brodmann areas 17/18 and 19 when localizing objects based on the SSD's input. Accordingly, although no study has investigated the neural structures that support natural human echolocation, functional neuroimaging research involving an echo-based SSD suggests the involvement of visual cortex. At the same time, it is important to recognize that the auditory signal used in natural human echolocation (i.e., the echo) is not only much weaker than that produced by the echo-based SSD employed in [10], but also that the process of natural echolocation differs from the SSD. In particular, unlike the echo-based SSD, natural human echolocation involves the comparison of a self-generated sound to that of its returning echo [11]. It is therefore unclear if the same neural structures that are recruited during the use of an echo-based SSD are also recruited during natural human echolocation. The present study was designed to investigate which brain areas mediate natural human echolocation. More specifically, we created auditory stimuli that allowed us to identify those brain areas that responded only to the echoes within a train of echolocation sounds.

Two blind skilled echolocators participated in the current study. Participant EB (43 years at time of testing) had partial vision up to 13 months of age. At 13 months, his eyes were removed due to retinoblastoma (early onset blindness). Participant LB (27 years at time of testing) lost vision at age 14 years due to optic nerve atrophy (late onset blindness). Both were right-handed, had normal hearing and normal auditory source localization abilities (Figure S1; Audiology Report S1; for samples of sounds used during source localization listen to Sound S3 and Sound S4). Both EB and LB use echolocation on a daily basis, enabling them to explore cities during travelling and to hike, mountain bike or play basketball. Two non-echolocating, right-handed sighted males, C1 and C2, were run as sex and age-matched fMRI controls for EB and LB, respectively. There is evidence that blind people, even when they do not consciously echolocate, are more sensitive to echoes than sighted people [12]. This might pose a challenge when comparing the brain activation of blind echolocators with the brain activation of blind self-proclaimed non-echolocators. For this reason, we decided to use sighted self-proclaimed non-echolocators as control participants.

The data show that the presence of echoes within a train of complex sounds increases BOLD signal in calcarine cortex in both EB and LB. This increase in activity in calcarine cortex is absent in C1 and C2. Importantly, the presence of echoes within a train of complex sounds does not lead to an increase in BOLD signal in auditory cortex in any of the four participants. This finding suggests that brain structures that process visual information in sighted people process echo information in blind echolocation experts.

## Results

### Validation of the Echolocation Stimuli

To overcome the difficulties posed by studying echolocation in an MRI environment (i.e., hearing protection must be worn, head and mouth movements must be minimized, etc.), a passive listening paradigm was adopted whereby the echolocation clicks and their echoes were pre-recorded in the listener's ears (Figure 1D) and then presented via fMRI compatible insert earphones (Figure 1E). To test the validity of this paradigm, a direct behavioral comparison between active echolocation and passive listening was conducted using an angular position discrimination task, in which EB and LB discriminated the angular position of a test pole with respect to straight ahead (Figure 1B). The results of this test are illustrated in Figure 1C. It is evident from the data that EB and LB can determine the angular position of the pole in both active and passive echolocation tasks (for samples of sounds used during angular position discrimination through passive listening listen to Sound S5 and Sound S6). For EB, thresholds are very low (approx. 3°) and performance in active and passive tasks is the same. Thus, EB can reliably distinguish a 3° difference in the position of the test pole away from straight ahead, even when listening only to recordings of echolocation sounds. For LB, thresholds are generally higher than for EB and performance in the active task (threshold approx. 9°) is better than in the passive task (threshold approx. 22°). With regard to bias, EB is unbiased (red line at zero), but LB tends to judge test locations to be to the left of the straight ahead (red line shifted to the right). This means, that LB's subjective straight ahead is shifted to the right. In summary, the data show that during active echolocation, both EB and LB resolved the angular position of a sound reflecting surface with high precision. This was expected based on what EB and LB do in everyday life. In addition, the data show that during passive listening, LB's precision was somewhat reduced, but EB's performance was unaffected, reflecting perhaps his greater experience with echolocation and/or the fact that he was blinded early in life. In any case, we felt confident that passive listening was a feasible paradigm to probe the neural substrates of echolocation in the scanner.

To obtain stimuli that would elicit strong echolocation percepts, we recorded echolocation clicks and echoes from EB and LB outside of the MRI under three scenarios: i) as they sat in an anechoic chamber in front of a concave or flat surface that was placed 40 cm in front of them and 20° to the left or right (for examples of sounds used during the experiment listen to Sound S7 and Sound S8); ii) as they sat in an anechoic chamber in front of a concave surface placed 40 cm in front with either the head held stationary or the head moving (when recordings of the latter were played back to EB and LB, they described a percept of a surface in motion; for examples of sounds used during the experiment listen to Sound S9, Sound S10 and Sound S11); and iii) as they stood outdoors in front of a tree, or a car, or a lamp post. We also created control sounds for the outdoor recordings, which contained the same background sounds and clicks, but no click echoes. Thus, outdoor control sounds were yoked to the outdoor echolocation sounds, but they did not contain the click's echoes (for examples of sounds used during the experiment listen to Sound S12 and Sound S13). Behavioral testing demonstrated that, when presented with the recordings from the anechoic chamber, EB was able to determine the shape, movement and location of surfaces with near perfect accuracy, whereas LB was less accurate at the shape and movement task and in fact performed at chance levels on the localization task (Figure 1F). Finally, when presented with the outdoor echolocation recordings both EB and LB readily distinguished control sounds from echolocation sounds and they identified objects well above chance levels. In addition, both echolocators performed equally well when listening to outdoor recordings of the other person as compared to their own (Figure 1G). Control participants C1 and C2 had trained with the echolocation stimuli of EB and LB prior to testing. Both control participants performed at chance levels for shape and location classification, but well above chance for movement classification (Figure 1F). Upon questioning, both C1 and C2 stated that clicks in ‘moving’ stimuli had a slightly more regular rhythm (compare Sound S9 and Sound S10 to Sound S11). However, both C1 and C2 maintained that they had not perceived any kind of movement in those recordings. When C1 and C2 were presented with outdoor recordings they could distinguish echolocation sounds from control sounds, but they were unable to identify objects (Figure 1G). Upon questioning, C1 and C2 reported that echolocation and control stimuli sounded ‘somehow different’, but they could not pinpoint the nature of this difference (compare Sound S12 and Sound S13). Both C1 and C2 said that they had not perceived any objects in the recordings. For more detailed results, including sample sizes, see Table S1 and Table S2.

### Brain activation

#### Cerebral Cortex

**Figure 2:**
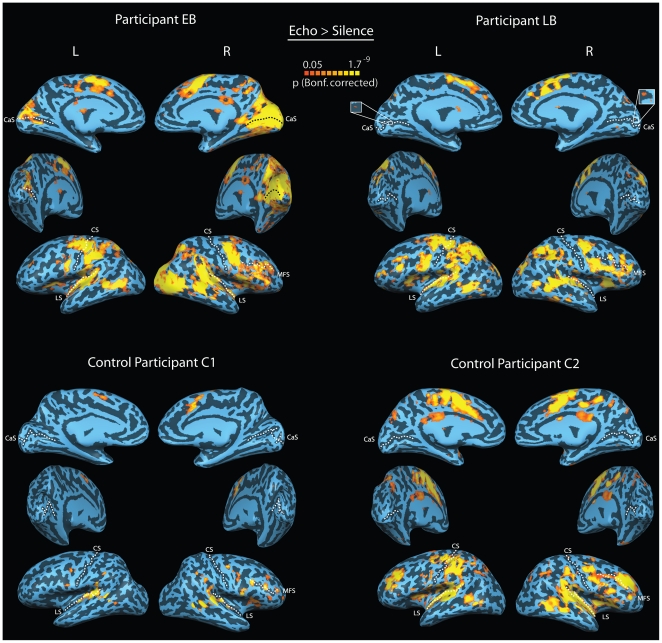
BOLD activity projected on participants reconstructed and partially inflated cortical surface. Concavities and convexities are colored dark and light, respectively. CS-central sulcus, CaS-calcarine sulcus, LS- lateral sulcus, MFS – middle frontal sulcus. Top panel: BOLD activity while EB and LB listened to recordings of their own echolocation sounds that had been made in an anechoic chamber and judged the location (left vs. right), shape (concave vs. flat) or stability (moving vs. stationary) of the sound reflecting surface (see Figure 1F for behavioral results). Bottom Panel: BOLD activity while C1 and C2 listened to recordings they had trained with, i.e. EB and LB's echolocation sounds, respectively. Just as EB and LB, C1 and C2 judged the location (left vs. right), shape (concave vs. flat) or stability (moving vs. stationary) of the sound reflecting surface (see Figure 1F for behavioral results). Both EB and LB, but not C1 or C2, show reliable BOLD activity in calcarine sulcus, typically associated with the processing of visual stimuli. EB shows more BOLD activity in calcarine sulcus than LB. All subjects (except C2) also show BOLD activity along the central sulcus (i.e. Motor Cortex) of the left hemisphere, most likely due to the response related right-hand button press. All subjects also show BOLD activity in the lateral sulcus (i.e. Auditory Complex) of the left and right hemispheres and adjacent and inferior to the right medial frontal sulcus. The former likely reflects the auditory nature of the stimuli. The latter most likely reflects the involvement of higher order cognitive and executive control processes during task performance.

Functional MRI revealed reliable blood-oxygen-level dependent (BOLD) activity in auditory cortex as well as in the calcarine sulcus and surrounding regions of “visual” cortex in EB and LB when they listened to recordings of their echolocation clicks and echoes, as compared to silence (Figure 2, top). EB showed stronger activity in the calcarine cortex than did LB, which could reflect EB's much longer use of echolocation and/or his more reliable performance in passive echolocation tasks. Activity in calcarine cortex was entirely absent in C1 and C2 when they listened to the echolocation recordings of EB and LB, although both control subjects showed robust activity in auditory cortex (Figure 2, bottom). This pattern of results was expected based on previous experiments that have measured brain activation in blind and sighted people in response to auditory stimulation as compared to silence [13]–[15].

**Figure 3:**
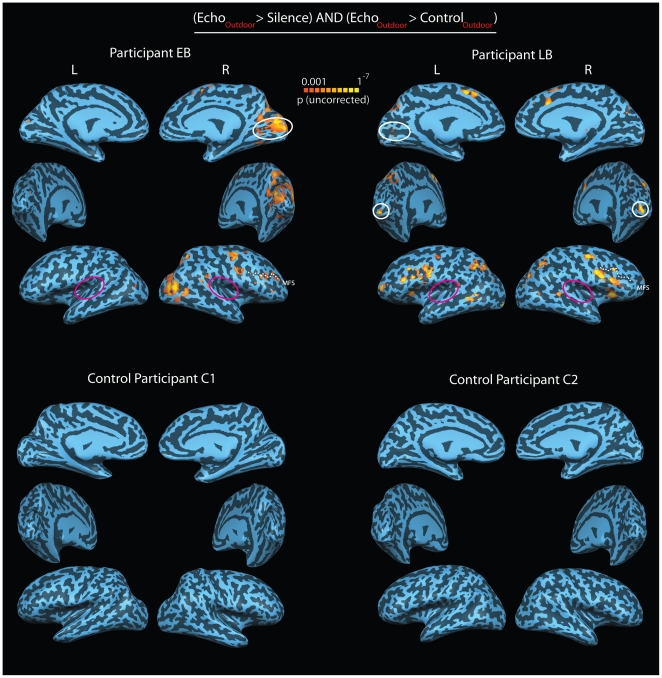
BOLD activity projected on participants reconstructed and partially inflated cortical surface. Marking of cortical surfaces and abbreviations as in Figure 2. Top panel: Contrast between activations for outdoor recordings containing echoes from objects and recordings that did not contain such echoes for EB and LB. During the experiment EB and LB listened to outdoor scene recordings and judged whether the recording contained echoes reflected from a car, tree or pole or no object echoes at all. Each participant listened to recordings of his own clicks and echoes as well as to recordings of the other person (see Figure 1G for behavioral results; for example sounds listen to Sound S12 and Sound S13). Bottom panel: Contrast between activations for outdoor recordings containing echoes from objects and recordings that did not contain such echoes for C1 and C2. The task was the same as for EB and LB and each participant listened to recordings they had trained with as well as to the recordings of the other person, e.g. C1 listened to both EB's and LB's recordings (see Figure 1G for behavioral results). It is evident that both EB and LB, but not C1 or C2, show increased BOLD activity in the calcarine sulcus for recordings that contain echoes (highlighted in white). EB mainly shows increased activity in the calcarine sulcus of the right hemisphere, whereas LB shows activity at the apex of the occipital lobes of the right and left hemisphere, as well as in the calcarine sulcus of the left hemisphere. In addition, both EB and LB, but not C1 or C2, show an increase in BOLD activity in along the medial frontal sulcus. This result most likely reflects the involvement of higher order cognitive and executive control processes during echolocation. There is no difference in BOLD activity along the lateral sulcus for any participant, i.e. Auditory Complex (highlighted in magenta). This result was expected because the Echo stimuli and the Control stimuli had been designed in a way that minimized any spectral, temporal or intensity differences. No BOLD activity differences were found when activations for EB's recordings were contrasted with activations for LB's recordings.

Remarkably, however, when we compared BOLD activation to outdoor recordings that contained click echoes with activation to outdoor recordings without echoes, activity disappeared in EB and LB's auditory cortex, but remained in calcarine cortex (Figure 3, top). Again, the activation in the calcarine cortex was more evident in EB than it was in LB. The results were quite different for the control participants. When we contrasted BOLD activity related to outdoor recordings that contained click echoes with those that did not, neither C1 nor C2 showed any differential activation in any region of their brains (Figure 3, bottom). The results also hold at a more liberal statistical threshold (Figure S2).

The lack of any difference in activity in auditory cortex in all the participants for the contrast between outdoor recordings with and without echoes was not unexpected, because we had created echolocation and control stimuli so that the acoustic differences were minimal and the only difference was the presence or absence of very faint echoes (Sound S12 vs. Sound S13). In addition, the environmental background sounds that were contained in both outdoor echolocation and outdoor control recordings made both kinds of stimuli meaningful and interesting to all participants. This, however, makes the increased BOLD activity in the calcarine cortex and other occipital cortical regions in EB and LB during echolocation all the more remarkable. It implies that the presence of the low-amplitude echoes activates ‘visual’ cortex in the blind participants (particularly in EB), without any detectable activation in auditory cortex. Of course, when we compared activation associated with both the outdoor echolocation and control recordings as compared to silence, there was robust activation in auditory cortex in both the blind and the sighted participants (Figure S3).

**Figure 4:**
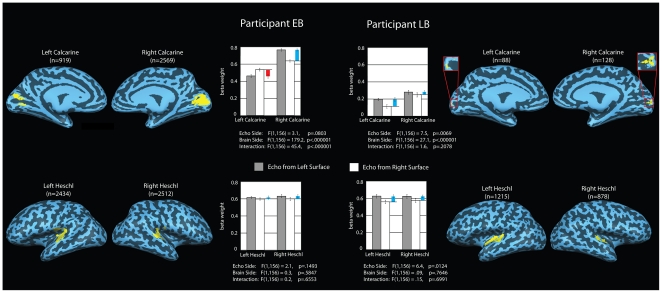
Results of the analysis of contralateral preference for EB and LB. Regions of interest (ROI) were defined based on anatomical and functional criteria. For illustration purposes, we show projections of ROI on the partially inflated cortical surfaces. However, all statistical analyses were performed in volume space. Bar graphs indicate beta values for the various ROIs. Gray and white bars indicate beta weights for ‘echo from surface on left’ and ‘echo from surface on right’, respectively, averaged across voxels within each ROI. Colored bars denote the difference between beta weights within each brain side (red bars indicate higher beta values for ‘echo from surface on right’; blue bars the reverse). Error bars denote SEM. To determine if activity during echolocation exhibits a contralateral preference, we applied independent measures ANOVA to the beta weights with ‘echo side’ (i.e. ‘echo from surface on left’ vs. ‘echo from surface on right’) and ‘brain side’ (e.g. ‘left calcarine’ vs. ‘right calcarine’) as factors to each ROI. ANOVA results are summarized below each bar graph. Results show that activity in calcarine cortex exhibits contralateral preference for EB (significant interaction effect), but not LB. Activity in auditory cortex shows neither contra- nor ipsilateral preference in either subject. For both EB and LB, beta values in the right calcarine exceed those in the left calcarine (main effect of ‘brain side’).

Given the echo related activation of calcarine cortex in both EB and LB, the question arises as to whether the echo related activity in calcarine cortex shows a contralateral preference – as is the case for light related activity in calcarine cortex in the sighted brain. To test this, we performed a region of interest analysis that compared BOLD activity in left and right calcarine in response to echolocation stimuli that contained echoes from surfaces located on the left or right side of space. For comparison, we also applied this analysis to the left and right auditory cortex. Previous fMRI research has shown a contralateral bias in auditory cortex for monoaural stimulation [16]–[18]. But to date, fMRI research has not been able to detect a contralateral bias with binaural stimulation, even though subjects may report hearing the sound source to be lateralized to the left or right, e.g. [18]. In short, we would not expect our ROI analysis to reveal a contralateral bias in auditory cortex. The results of our ROI analyses are shown in Figure 4. As can be seen, activity in calcarine cortex exhibited a contralateral bias in EB, but not LB (Figure 4, bottom). In other words, EB's calcarine cortex showed the same kind of contralateral bias for echoes as the calcarine cortex in sighted people shows for light. As expected, there was no evidence for contralateral bias in auditory cortex in either EB or LB (Figure 4, bottom).

**Figure 5:**
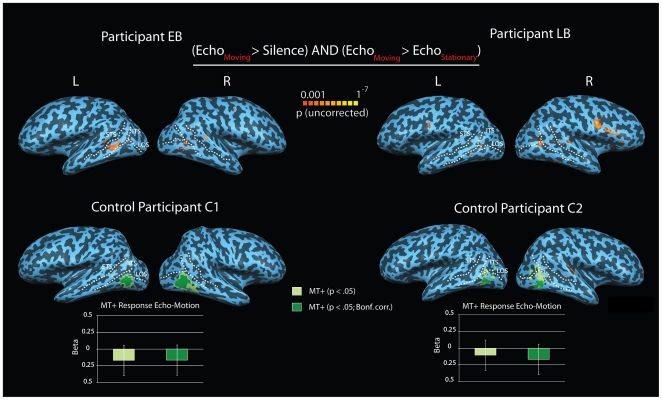
BOLD activity projected on participants reconstructed and partially inflated cortical surface. Concavities and convexities are colored dark and light, respectively. STS-superior temporal sulcus, ITS -inferior temporal sulcus, LOS – lateral occipital sulcus. **Top Panel:** BOLD activity related to recordings of echolocation sounds conveying movement to EB and LB. Both EB and LB show significant activity in regions adjacent and inferior to the ITS/LOS junction, that are typically involved in motion processing. **Bottom Panel:** BOLD activity in C1 and C2's brain related to recordings of echolocation sounds that convey movement to EB and LB. Even though C1 and C2 could reliably classify echolocation sounds as ‘moving’ or ‘stationary’, they reported to not perceive any sense of movement. Also shown are areas sensitive to visual motion (area MT+) functionally defined at different significance levels (p<.05: light green or p<.05 Bonf. Corrected: dark green). Bar graphs show beta weights (+/− SEM) obtained from a region of interest analysis applied to areas MT+ (contrast: Echo_Moving_>Echo_Stationary_). Bar color denotes the MT+ used for the ROI analysis (i.e. MT+ defined at p<.05: light green, or p<.05; Bonf. Corrected: dark green). In contrast to EB and LB, neither C1 nor C2 show increased BOLD activity in regions adjacent and inferior to the ITS/LOS junction for the contrast between ‘moving’ and ‘stationary’ echolocation stimuli, even at more liberal statistical thresholds (see Figure S4). The statistically more powerful region of interest analysis applied to area MT+ was not significant either, i.e. SEM error bars (and therefore any confidence interval) include zero (see also Table S3).

Finally, we also examined BOLD activity related to echolocation stimuli that conveyed object movement with activity related to stimuli that did not convey such movement in both the blind and the sighted participants. Both EB and LB showed activity in areas of the temporal lobe commonly associated with motion processing (Figure 5 top). This activity was absent in the control participants (Figure 5, bottom), who also did not perceive any sense of movement. The results also hold at a more liberal statistical threshold (see Figure S4). Also a more powerful region of interest analysis for C1 and C2, in which we analyzed the response to echolocation motion stimuli within functionally defined visual motion areas MT+, did not reveal any significant activation (Figure 5, bottom; Table S3).

The comparison between concave vs. flat conditions, as well as the comparison between tree vs. car vs. pole did not reveal significant differences. It is evident from the behavioural data, that EB and LB certainly perceived these conditions as different; so at some level, there must be a difference in neural activity. It is likely that the temporal and spatial resolution of our paradigm was not able to detect these differences.

#### Cerebellum

It is well established that the cerebellum is involved in the control and coordination of movement, and there is also mounting evidence that the cerebellum may be involved in higher order cognitive function (for reviews see [19]–[24]). Recently, it has also been suggested that the cerebellum is involved in purely sensory tasks, such as visual and auditory motion perception [25]. Consistent with the idea that the cerebellum might be involved in non-motor functions in general, and sensory processing in particular, we also observed significant BOLD activity in the cerebellum in both the blind and the sighted participants in our experiments. We identified and labeled cerebellar structures based on anatomical landmarks and the nomenclature developed by [26].

**Figure 6:**
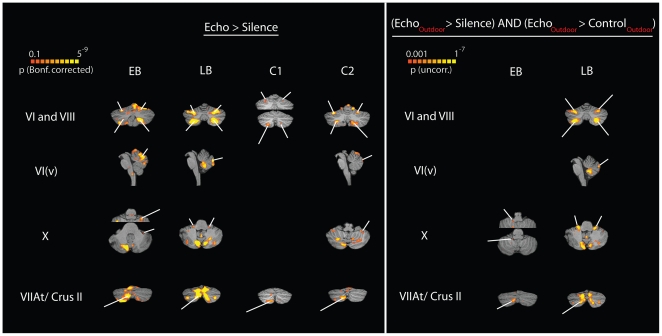
BOLD activity in the cerebellum. Data are shown in neurological convention, i.e. left is left. Activity in the cerebellum was analyzed in stereotaxic space [49]. To evaluate significance of activity we used the same voxelwise significance thresholds as for cortical surface analyses for each participant. However, because the number of voxels in volume space differed from the number of vertices in surface space for each participant, the Bonferroni corrected significance level differs between cortex and cerebellum (compare Figure 2). To increase accuracy, cerebellar structures for each participant were identified based on anatomical landmarks. Structures were labeled according to the nomenclature developed by [26]. **Left panel:** BOLD activity while participants listened to recordings of echolocation sounds that had been made in an anechoic chamber and judged the location (left vs. right), shape (concave vs. flat) or stability (moving vs. stationary) of the sound reflecting surface (see Figure 1F for behavioral results). **Right Panel:** Contrast between BOLD activations for recordings containing echoes from objects and recordings that did not contain such echoes. Data are not shown if no significant activity was found (empty cells in table).

When EB and LB listened to recordings of their echolocation clicks and echoes, as compared to silence, they both showed significant BOLD activity in lobules VI and VIII (Figure 6, left). A similar pattern was observed in the two sighted participants (Figure 6, left). In other words, lobules VI and VIII appeared to be more active when all our participants listened to auditory stimuli as compared to silence. This pattern of activity is generally consistent with results that link activity in lobules VI and VIII to auditory sensory processing [25]. We also found robust activation in left lobule VIIAt/Crus II in all participants (Figure 6, left). To date, lobule VIIAt/Crus II has not been implicated in sensory processing, but it has been suggested that it is part of a non-motor loop involving Brodmann area 46 in prefrontal cortex [24]. Consistent with this idea, the activation in left lobule VIIAt/Crus II coincides with activity adjacent and inferior to right medial frontal sulcus in all participants (compare Figure 2). Finally, both EB and LB showed robust activation in vermal lobule VI and lobule X, both of which have been linked to visual sensory processing [25]. Interestingly, however, C2 also shows activity in vermal lobule VI and close to lobule X. In summary, for the comparison of echolocation to silence, we found reliable activation in the cerebellum, but this activation did not clearly distinguish between EB and LB on the one hand, and C1 and C2 on the other.

The result was different, however, when we compared BOLD activation to outdoor recordings that contained click echoes with activation to outdoor recordings that did not contain echoes. Specifically, this analysis did not reveal any differential activity anywhere in the cerebellum for the two sighted control subjects C1 and C2. In contrast, for both EB and LB, this analysis revealed differential activity in lobule X and lobule VIIAt/Crus II (Figure 6, right). Again, activity in left lobule VIIAt/Crus II coincides with activity adjacent and inferior to the right middle frontal sulcus in both EB and LB (compare Figure 3). In addition, for LB only, this analysis also revealed differential activity in vermal lobule VI and lobules VI and VIII.

Of course, when we compared activation associated with both the outdoor echolocation and control recordings as compared to silence, the pattern of activity in the cerebellum was very similar to when we compared activation associated with echolocation sounds to activation associated with silence (Figure S5).

The comparison between concave vs. flat conditions, as well as the comparison between tree vs. car vs. pole did not reveal significant differences

## Discussion

Here we show that two blind individuals can use echolocation to determine the shape, motion and location of objects with great accuracy, even when only listening passively to echolocation sounds that were recorded earlier. When these recordings were presented during fMRI scanning, we found that ‘visual’ cortex was strongly activated in one early blind participant (EB) and to a lesser degree in one late blind participant (LB). Most remarkably, the comparison of brain activity during sounds that contained echoes with brain activity during control sounds that did not contain echoes revealed echo related activity in calcarine, but not auditory cortex.

The question arises if the activity that we observe in calcarine cortex is truly related to echolocation, or if it is simply due to the fact that EB and LB are blind. Blindness can result in re-organization of many brain areas, including but not limited to visual, auditory and somatosensory cortex and subcortical structures, even though the underlying mechanism and exact nature of the changes are still unclear [13]–[15], [27]–[32]. Based on the existing literature, therefore, it is not surprising to see activity in visual cortex in response to auditory stimuli in EB and LB. However, support for an interpretation of the activation in terms of echolocation, but not blindness per se, is provided by the outdoor scenes experiment, in which we see differential activation in calcarine cortex in EB and LB, but not in auditory cortex when echoes are present (or not) in the outdoor sounds (Figure 3). In this regard our data go beyond ‘classical’ cross-modal results that show co-activation of visual cortex and areas primarily sensitive to the stimulus (i.e. primary auditory or somatosensory cortex). In a related point, we want to emphasize that the differences in the level of activation in the visual areas of EB's and LB's brains could have arisen for a number of reasons. First, there might be differences in cortical development in the two individuals; after all, EB lost his sight much earlier than LB. Second, EB started using echolocation as a small child and has used it longer than LB. A consequence of this might be that EB creates a more vivid representation of the spatial scene from click-echoes. Third, EB performed better in the passive-listening paradigm than LB even though this difference was reduced for ‘outdoor’ sound recordings. But of course, any combination of all these factors could account for the differences in the activity in visual areas we observed in these two individuals.

It would be useful in future neuroimaging studies of echolocation to include sighted people who have been trained to echolocate, or blind people who have a ‘regular’ sensitivity to echoes. With respect to the latter, there is evidence that blind people, even when they do not consciously echolocate, are more sensitive to echoes than sighted people [12], and this might pose a challenge when comparing the brain activation of self-proclaimed echolocators to the brain activation of self-proclaimed non-echolocators who are also blind. In any case, the comparison we draw here (i.e. between blind echolocators and sighted non-echolocators) is insightful, because it highlights the involvement of visual rather than auditory cortex in the processing of echoes.

The patterns of activation observed in their brains might shed some light on the possible role that sensory deprivation plays in the recruitment of visual cortex during echolocation in the blind. On the behavioural level, of course, sighted people's echolocation abilities have been repeatedly shown to be inferior to those of blind people (for reviews see [1]–[3]). There are various reasons why this is the case. One possibility is that blind people use echolocation on a daily basis and therefore acquire a higher skill level through practice. Another possibility might be that blind people have better hearing abilities which may also make them better at echolocation, e.g. [33], [34]. Our current data suggest that hearing ability is not a variable, because both EB and LB performed within the normal range on standard hearing and source localization tests (Figure S1; Audiology Report S1). Furthermore, we also saw no obvious differences in activation in auditory cortex between EB and LB or between these two individuals and the control participants (Figure 2, Figure S3). It cannot be ruled out, however, that the tests and comparisons we used are not suitable for detecting the auditory abilities that may underlie superior echolocation performance. Finally, it is also possible that sighted individuals might simply be at a disadvantage in acquiring echolocation skills, because echolocation and vision compete for neural resources. Clearly, more investigations of human echolocation are needed on the behavioural, computational, and neural level, to uncover how echolocation works, how it is acquired and which neural processes are involved.

It is important to emphasize that the use of echolocation in the blind goes well beyond localizing objects in the environment. The experts we studied were also able to use echolocation to perceive object shape and motion – and even object identity. In addition, they were able to use passive listening with 10-kHz cut-off to do these kinds of tasks – which made it possible for us to probe neural substrates of their abilities. Clearly more work is needed comparing performance with active and passive echolocation across a range of different tasks – where the available frequency ranges in both conditions are systematically varied.

It could be argued that the contralateral bias that we observed in EB's calcarine cortex reflects differences in spatial attention between the two conditions. Effects of attention on brain activity have been shown for visual [35], as well as other cortical areas, including auditory cortices, e.g. [17], [36]. Thus, although we cannot rule out this explanation, it would still be remarkable that EB, who lost his eyes when he was 13 months of age, would show attentional modulation of the calcarine cortex, but not the auditory cortex – and would do this in a contralateral fashion.

Both EB and LB show BOLD activity in temporal cortical regions typically devoted to motion processing, but this activity is absent in C1 and C2. In a similar fashion, both EB and LB reported to perceive motion, but this percept was absent in C1 and C2. Thus, we see good correspondence in terms of brain activity and perception. The question remains, however, as to what the ‘preferred modality’ of the neurons is that are active in EB and LB when they perceive motion using echolocation. Neurons adjacent and inferior to the ITS/LOS junction are sensitive to both visual and auditory motion as determined with functional localization techniques [37]. Sighted individuals typically show a modality specific cortical organization, such that neurons that are sensitive to visual motion (i.e. area MT+) are located adjacent but posterior to neurons that are sensitive to auditory motion [37]. In contrast, individuals who regained vision at a later point in their life (i.e. late onset sight recovery) show cortical organization that is not modality specific, such that visual and auditory motion areas largely overlap [37]. Finally, neurons in and around visual motion area MT+ may also respond to tactile motion, even though it remains to be determined to what degree this activity is potentially mediated by visual imagery [38]–[40]. Future research is needed to investigate how neurons that are active during echolocation motion correspond to visual motion area MT+ in sighted people.

An obvious question that arises from our findings is what function calcarine cortex might serve during echolocation. One possibility is that it is involved in the comparison between outgoing source sound (e.g. mouth click) and incoming echo. This explanation seems unlikely, however, because if the calcarine computed a comparison between outgoing source sound and incoming echo, it would also compute that comparison in the absence of echoes. If that were the case, however, we would expect the calcarine to be equally active in the presence and the absence of echoes – provided the corresponding clicks were present. The pattern of activity we found in EB and LB does not support this interpretation (Figure 3). An alternative, and perhaps more plausible, explanation is that calcarine cortex performs some sort of spatial computation that uses input from the processing of echolocation sounds that was carried out elsewhere, most likely in brain areas devoted to auditory processing. In this case, one would expect calcarine cortex to be more active in the presence than in the absence of echoes, because the trains of sounds with echoes contain more spatial information than those without echoes. The activity patterns we found in EB and LB would certainly support this interpretation (Figure 3). We are not the first to propose that visual cortex could potentially subserve ‘supra-modal’ spatial functions after loss of visual sensory input [41]. Recently, a similar supra-modal spatial function has also been suggested for certain parts of auditory cortex after loss of auditory sensory input [42]. Again, future research is needed to determine exactly how activity in calcarine cortex mediates echolocation.

The cerebellar structures linked to visual sensory processing [25] also appear to play a role in echolocation in the blind. In particular, we found that lobule X is more active in both EB and LB during echolocation than during control sounds. Thus, the arguments discussed above for potential function of calcarine cortex during echolocation also apply to lobule X.

In addition to lobule X, we also found activity in left lobule VIIAt/Crus II during echolocation. Since this part of the cerebellum is involved in a non-motor loop involving Brodmann area 46 in pre-frontal cortex [24], the co-activation that we see in this part of the cerebellum and in cortex adjacent and inferior to the right middle frontal sulcus makes sense. As a caveat, we want to note however, that we cannot be certain that the activity we found adjacent and inferior to the middle frontal sulcus actually corresponds to activity in Brodmann area 46, because there is natural variability in the anatomical location of Brodmann area 46 in the human brain [43]. In any event, we suggest that the activation of right middle prefrontal cortex and left cerebellar lobule VIIAt/Crus II most likely reflects the involvement of cognitive and executive control processes that are non-echolocation specific. This hypothesis is supported by the fact that we also saw activity in these brain areas in C1 and C2. It is unlikely that this activity reflects motor imagery or the activation of a ‘click motor-scheme’ during the passive listening paradigm, because the click sound was the same between outdoor echo and outdoor control stimuli where only the echo was missing.

### Conclusion

The current study is the first to investigate which brain areas potentially underlie natural echolocation in early- and late-blind people (EB and LB). In EB, we found robust echolocation-specific activity in calcarine cortex – but not in auditory cortex. A similar pattern was observed in LB, but the activity in the calcarine cortex was not as extensive. We also found that the calcarine activity was greater for echoes reflected from surfaces located in contralateral space in EB but not LB. Our findings also shed new light on how the cerebellum might be involved in sensory processing. In addition, our study introduced novel methodology that can be used in future experiments on echolocation.

From a more applied point of view, our data clearly show that EB and LB use echolocation in a way that seems uncannily similar to vision. In this way, our study shows that echolocation can provide blind people with a high degree of independence and self-reliance in their daily life. This has broad practical implications in that echolocation is a trainable skill that can potentially offer powerful and liberating opportunities for blind and vision-impaired people.

## Materials and Methods

All testing procedures were approved by the ethics board at the University of Western Ontario, and participants gave written informed consent prior to testing. The consent form was read to participants, and the location to sign was indicated manually.

Software used to conduct testing was programmed using Psychophysics toolbox 2.54 [44], Matlab7 (R14, The Mathworks) and C/C++. fMRI data were analyzed using Brain Voyager QX version 2.1 (Brain Innovation, Maastricht, The Netherlands) and Matlab R14 (The MathWorks, Natick, MA, USA). Sound editing was performed with Adobe Audition version 1.5 software (Adobe Systems, San Jose, CA, USA). Sound equalization was performed with filters provided by the headphone manufacturer (Sensimetrics, Malden, MA, USA).

### fMRI Data Acquisition

All imaging was performed at the Robarts Research Institute (London, Ontario, Canada) on a 3-Tesla, whole-body MRI system (Magnetom Tim Trio; Siemens, Erlangen, Germany) using a 32-channel head coil.

#### Setup and Scanning Parameters

*fMRI Echolocation:* Audio stimuli were delivered over MRI-compatible insert earphones (Sensimetrics, Malden, MA, USA, Model S-14). Earphones were encased in replaceable foam tips that provided a 20–40 dB attenuation level (information provided by the manufacturer). Further sound attenuation was attained by placing foam inserts between the head rest and the listener's ears. To minimize background noise, the MRI bore's circulatory air fan was turned off during experimental runs. A single-shot gradient echo-planar pulse sequence in combination with a sparse-sampling design [45] was used for functional image acquisition. Repetition time [TR] was 14 s (12 s silent gap+2 s slice acquisition). We used a FOV of 211 mm and 64×64 matrix size, which led to in-slice resolution of 3.3×3.3 mm. Slice thickness was 3.5 mm and we acquired 38 contiguous axial slices covering the whole brain (including cerebellum) in ascending interleaved order. Echo time [TE] was 30 ms and Flip-Angle [FA] was 78°.

*fMRI MT+ Localizer (C1 and C2 Only):* Visual stimuli were viewed through a front-surface mirror mounted on top of the head coil and were projected with an LCD projector (AVOTEC Silent Vision Model 6011, Avotec, FL, USA) on a rear-projection screen located behind the head-coil in the bore. fMRI scanning parameters were the same as the echolocation experiments, with exception of a 2 s TR related to the continuous scanning procedure.

*Anatomical Images:* Anatomical images of the whole brain were acquired at a resolution of 1×1×1 mm using an optimized sequence (MPRAGE).

#### Functional Paradigms

*Shape/Location:* Each run contained silent baseline and experimental trials. Experimental trials began with a pre-recorded spoken instruction (i.e., “shape” or “location”) indicating which attribute the listener should attend to from the echo. Total time including the brief silent gap that followed the instruction was 1 s. Next, 10 s of echolocation stimuli were presented. Since stimuli were shorter than 10 s (see experimental stimuli) the sound was played in a loop. This was followed by a 200 ms 1000 Hz tone. The participant was instructed to indicate his response with a key press after he heard the tone (see behavioral paradigm below). Functional scans started 12 s after the run had started and lasted 2 s. The next trial started after scanning had ended. Silent baseline trials differed from experimental trials in that the 2 s functional scan occurred after 12 s of silence. No cues were provided and no key-presses were produced. Trials were counterbalanced such that a silent trial always preceded two experimental trials and that experimental trials occurred in alternating order (i.e. shape-location followed location-shape and vice versa). Each run began and ended with a silent baseline trial. The total number of trials in each run was 25 (8 shape, 8 location and 9 silent) and each run lasted 25×14 s. Each participant performed 5 runs.

*Motion:* Motion experiment runs were the same as in the Shape/Location experiments with the exception that no cue was presented prior to the echolocation sounds, thus making the echolocation stimuli duration 11 s. Trials were counterbalanced such that a silent trial always preceded two experimental trials and that experimental trials occurred in alternating order (i.e. stationary-moving followed moving-stationary and vice versa). Each participant performed 5 runs.

*Outdoor Scenes:* Outdoor Scene runs were similar to those in the motion experiment. Stimuli were played for 11 s. Participants listened to scene echolocation recordings from both persons (thus, four different experimental conditions, i.e. EB-Echo, EB- Control, LB-Echo, LB-Control). Stimuli presentation order was balanced using a clustered Latin square design, such that each run contained four clusters, each cluster contained all 4 experimental conditions, and the order of conditions within each cluster was chosen such that every condition was preceded by every other condition in a run. A cluster was always preceded by a silent baseline trial and each run began and ended with a silent baseline trial. Thus, there were 21 trials per run (5 silent+4×4 experimental) and the duration of each run was 21×14 s. Each participant performed 6 runs.

*MT+ Localizer (C1 and C2 Only):*We employed a standard MT+ localizer paradigm that displayed white dots that were either stationary or moved in smooth linear motion in front of a black background. See Methods S1 for more details.

#### Behavioral Paradigms

*Shape/Location:* The basic paradigm was a 1-interval-2-alternative forced choice (AFC) paradigm. The participant listened to the echolocation sound and, depending on the cue, judged the shape (concave vs. flat) or location (right vs. left) of the sound reflecting surface. The participant indicated his response on an MR compatible keypad by pressing the key located under his right index or middle finger, respectively.

*Motion:* The basic paradigm was a 1-interval-2-AFC paradigm. The participant listened to the echolocation sound and judged the motion (moving vs. stationary) of the sound reflecting surface as conveyed by the echo. As in the shape/location experiment, responses were collected with the same keypad and the participant indicated his response by pressing the key located under his right index or middle finger, respectively.

*Outdoor Scenes:* The basic paradigm was a 1-interval-4-AFC paradigm. The participant listened to the echolocation sound and judged whether the scene contained a car, a tree or a pole or no sound reflecting object at all (Control Sounds). The response in the Scenes experiment was obtained with the same keypad as in the other experiments and the participant pressed the key located under his right index, middle, ring and little finger to report ‘tree’, ‘pole’, ‘car’ and ‘nothing’, respectively.

#### Order of experiments

(see Methods S1).

### fMRI Data Analysis

Standard routines were employed for fMRI data pre-processing, coregistration and cortical surface reconstruction (see Methods S1).

#### Functional Analysis – Voxelwise

*BOLD activity related to echolocation as compared to silence:* To obtain activity related to echolocation processing as compared to a silent baseline for each participant, we applied a fixed effect GLM with the stick-predictor “Echo” to the z-transformed time courses of runs obtained in shape/location and motion experiments (10 runs per participant). To determine where BOLD activity during echolocation trials exceeded that during silent baseline trials, we isolated voxels where the beta value of the ‘Echo’ predictor was significantly larger than zero. The significance threshold for evaluation of results in volume space was set to 0.1 (Bonferroni corrected (BC) and taking into account all voxels in the functional volume) in order to remove obvious false positives (e.g., activations outside of the brain) while still showing positive activation in expected areas (i.e. in auditory cortex) (see Methods S1 for more details). As it turned out, a .1 (BC) threshold in volume space corresponded very closely to a .05 (BC) threshold in surface space for each participant. Hence, we applied a .05 threshold (BC) to the cortical data in surface space and a threshold of .1 (BC) to the cerebellum data in volume space.

*BOLD activity related to moving echoes:* To obtain activity related to processing of moving echoes as compared to stationary echoes for each participant, we applied a fixed effect GLM with stick-predictors “moving” and “stationary” to the z-transformed time courses of runs obtained in motion experiments (5 runs per participant). The GLM results were then subjected to a conjunction analysis, i.e. (moving>0) AND (moving>stationary), the significance threshold for which was set to 0.001 (voxelwise) for both surface and volume data. To increase power for our control participants we also used a threshold of p<.01.

*BOLD activity related to outdoor sounds:* To obtain activity related to processing of outdoor sounds, regardless of the presence of echoes (i.e. echolocation vs. Control sounds) or participant (i.e. EB or LB) for each participant, we applied a fixed effect GLM with four stick-predictors, i.e. “EB-Echo”, “EB-Control”, “LB-Echo” and “LB-Control” to the z-transformed time courses of runs obtained in scenes experiments (6 runs per participant). The GLM results were then subjected to a contrast (i.e., “EB-Echo”+“EB-Control”+“LB-Echo”+“LB-Control”) against zero. The significance threshold for this contrast was chosen as in “*echolocation as compared to silence*”.

*BOLD activity related to outdoor echolocation sounds as compared to outdoor control sounds:* To obtain activity related to processing of outdoor echolocation sounds as compared to outdoor control sounds, regardless of the participant (i.e. EB or LB), the results of the GLM as described in the previous paragraph were subjected to a conjunction analysis, i.e. (EB-Echo+LB-Echo)>0 AND (EB-Echo+LB-Echo)>(EB-control+LB-control). The significance threshold for this was set to 0.001 (voxelwise). To increase power for our control participants we also used a threshold of p<.01.

#### Functional Analysis – ROI

*ROI Selection for analysis of contralateral preference (EB and LB only):* ROIs were defined anatomically and functionally. Anatomically, we considered voxels only within and in close proximity to the left and right calcarine sulcus (ROI: left and right calcarine) and the left and right Heschl's gyrus (ROI: left and right Heschl's gyrus). To avoid ‘bleeding in of activity’ from the right to the left hemisphere, and vice versa, we defined a 6 mm voxel selection gap between left and right hemispheres for the ROI definition for the calcarine. Functionally, we considered only those voxels for which the contrast (Echo_Motion_+Echo_Stationary_)>0 was significant. The minimum threshold for statistical significance to select voxels in any ROI was p<.001 with a combined cluster-size threshold of 10 voxels. For various ROIs, however, we adopted more stringent levels of significance, either to shrink a large area of activity to a more localized cluster (e.g. for the right calcarine in EB) or in order to uniquely determine the source of activity. More details are provided in Methods S1. Importantly, in all cases we confirmed with additional statistical analyses that the results of our ROI analysis held regardless of ROI selection criteria.

*ROI Analysis of contralateral preference (EB and LB only):* To determine activity for echoes from objects located to the right or left side of space, regardless of task (i.e. shape or location) or surface shape (i.e. concave or flat), we applied a GLM with stick-predictors “left” and “right” to the time courses of runs obtained in shape/location experiments (5 runs per participant). Thus, data for functional ROI analysis were independent from data used for ROI selection. Predictors as well as the time course for each voxel were z-transformed before the analysis. It follows that beta values obtained from the GLM are equivalent to correlation coefficients. The GLM was run as a fixed effect model for each voxel inside each ROI and participant.

From this analysis we obtained a separate beta value for ‘left’ and ‘right’ predictors for each voxel. To determine if there was a right or left echo preference in the left or right portion of the calcarine sulcus or Heschl's gyrus, we subjected those beta values to an ANOVA with ‘brain side’ and ‘echo side’ as independent factors, separately for the calcarine sulcus and Heschl's gyrus. Technically, we could have used the number of beta values to determine error degrees of freedom (*df*) for each ANOVA, but this would have resulted in different *df* for the error terms (and thus differences in statistical power) between participants and ROIs. To avoid this, we determined *df* based on the number of times an event occurred. For example, in the calcarine, ‘left’ and ‘right’ events each occurred 40 times in the left and 40 times in the right hemisphere resulting in 160 independent events and 156 *df* for the error term to compute the ANOVA for the calcarine sulcus. The same applies to the ANOVA applied to Heschl's gyrus.

In this way we could use data obtained from all voxels inside each ROI to determine interaction effects between ‘brain side’ and ‘echo side’ for each participant. In contrast, a traditional ROI analysis averages across voxels before applying the GLM, such that interaction effects can only be computed when data from multiple participants is available.

*MT+ ROI Selection (C1 and C2 only):* First, we applied a fixed effect GLM to determine which voxels showed activity during a ‘moving’ visual stimulus. MT+ was then defined by selecting voxels posterior to the ITS/LOS junction for which the activity was significant. For selection we used both a liberal voxelwise p<.05 threshold and more conservative Bonferroni corrected p<.05 threshold, where the correction was computed based on all voxels in the functional volume. For more details and ROI MT+ coordinates see Methods S1and Table S4.

### Experimental Stimuli

#### Setup and Recording Procedure - Anechoic Chamber

With the exception of the outdoor recordings, all auditory stimuli were recorded in the Beltone Anechoic Chamber at the National Centre for Audiology in London, Ontario, Canada, that was equipped with a 125 Hz cut-off wedge system on the walls and ceiling, and a vinyl covered concrete floor. Ambient noise recordings indicated a background noise (i.e., ‘noise floor’) of 18.6 dBA. The participant was seated in the center of the room. For each recording trial, the experimenters placed an object at a desired position, and then retreated to the back of the chamber (approximately 1.5 m behind the participant) before instructing the participant to start producing echolocation clicks. High-quality stereo recordings of the entire sessions' audio were acquired with the in-ear microphones and saved for off-line editing. EB and LB participated in separate recording sessions, i.e. during any recording session three people were in the room (two experimenters and one participant).

*Shape/Location:* Two surfaces were used to generate recordings for the shape and location classification experiments. The first was a standard sized safety helmet, made from plastic, and positioned such that the helmet's inside faced the participant (*concave* surface). The second surface was a wooden 12 cm-cube with smooth paint finish, positioned such that one of the cube's flat sides faced the participant (*flat* surface). Objects were positioned at a distance of 40 cm from the seated listener, either 20° to the *left* or *right* of straight ahead. The height of the object was adjusted on a 0.5 cm diameter telescopic steel pole so as to create optimal echolocation conditions as indicated by each participant (i.e., typically at participant's mouth level or approximately 1.3 m above the floor). For each of the four conditions (concave or flat surface, positioned to the left or right) recordings were made as follows: First the surface was placed. Then, the participant (either EB or LB) produced at least 20 echolocation clicks with his head held stationary and straight ahead.

*Motion:* It is possible to mimic the echolocator's perception of a moving object by recording echolocation clicks from a head in different positions relative to a stationary object, and then playing these recordings back to an echolocator whose head is stationary. To create the perception of moving objects, we made audio recordings with a concave surface positioned to the left or right (as described for the shape/location experiment), but this time the participant made echolocation clicks with his head in different positions during clicking, rather than held stationary straight ahead. Several examples of these echolocation sequences were recorded for each object position and (i.e., 20° left or right). Each sequence contained 6–9 clicks. The participant started and ended each sequence with his head held straight ahead.

*Angular Position Discrimination (Passive Listening):*To create stimuli for the angular position discrimination via passive listening, a position marker (described in main text) was placed at a radial distance of 150 cm at various angular intervals around the participant (i.e. straight ahead and 36°, 27°, 18°, 16°, 14°, 12°, 10°, 8°, 6°, 4°, 2°, 1° to the left and right of the straight ahead). Then, the participant (either EB or LB) produced at least 20 echolocation clicks with his head held stationary and aimed straight ahead.

#### Setup and Recording Procedure - Outdoor Scenes

Stimulus recording for the Scenes experiments took place in a garden-style courtyard, approximately 40 m long by 20 m wide and surrounded by an elliptical driveway. Two thirds of the driveway was bordered by two-storey buildings (see Figure S6). Echolocation recordings were made while the participant made clicks in front of a sound reflecting object (i.e. a tree, lamp-post or car, see Figure S7). Recordings were made separately for each object and participant. Echolocation clicks were self-paced (SOA roughly 500 ms) with the participant sampling the object at slightly different head positions. Non-clicking, baseline audio recordings (approximately 15 s in duration) were made while the participant stood silently in front of each sound reflecting object. Again, recordings were made separately for each object and participant.

#### Sound Editing

*Shape/Location:* For the Shape/Location experiment, two unique click sequences were extracted from each of the 20 clicks that were produced in the anechoic chamber by each echolocator during each of the conditions (i.e., concave left, concave right, flat left and flat right). Each of these click sequences was approximately 5 s in duration, which, depending on the participant's clicking rate, resulted in sequences containing anywhere from 6–9 clicks. The total number of click sequences used in the Shape/Location experiment was 16 (4 conditions×2 echolocators×2 exemplars), 8 for each participant.

*Motion:* Four unique click sequences were produced for each condition in the Motion recording sessions (object left or right). All ‘moving’ head stimuli contained in between 6–9 clicks and had duration of approximately 5–6 s. ‘Stationary’ head stimuli (object left and object right) were taken from the Shape/Location experiment in which the echolocators had made clicking sounds at the same concave object located in the same left and right positions, but always with their head fixed and oriented straight-ahead. The total number of click sequences for the motion experiment was 32 (2 object positions×2 types of head motion (moving, stationary)×4 exemplars×2 echolocators), 16 for each participant. To match the number of stationary exemplars to the number of moving ones, each stationary exemplar had been duplicated once.

*Angular position discrimination (Passive Listening):*With respect to the Angular Position Discrimination recording sessions, two unique click sequences of exactly 6 clicks each were extracted for each of the 25 pole locations (see *Angular Position Discrimination*), summing to a total of 50 stimuli (25 pole locations×2 exemplars) for each echolocator.

*Outdoor Scenes:* Two unique 5 s exemplars were extracted from each of the ‘scenes’ recordings (i.e., the sequence of 20 clicks made in front of a car, tree, or pole by each echolocator). This provided 12 sound files (3 object scenes×2 echolocators×2 exemplars). Depending on the participant's clicking rate, each of these sound files contained anywhere between 6 and 12 clicks in those 5 s. To create the control stimuli, we took the non-clicking baseline audio recordings that were made as each echolocator silently stood in front of the three objects (car, tree and pole), and we extracted two unique 5 s recordings from each. This provided us with 12 sound files (3 object scenes×2 echolocators×2 exemplars) containing only background noises (i.e., distant traffic, wind, birds, etc.), but no clicks or click echoes. Next, the click sequences, but not the echoes associated with them, were copied from each of the corresponding echolocation sound files, and then overlayed onto the respective sound files containing just the background noise. More specifically, with the aid of a spectral waveform display (see for example Figure 1A), the initial 10–20 ms burst of energy associated with the onset of each mouth-generated click was selected by hand from the left channel, being careful to avoid including any energy associated with click echoes. Each copy of these click waveforms was then overlayed in both left and right channels of the corresponding background noise file, at the precise time point that it had been copied from. This was carried out for every click in each of the 12 echolocation sound files. In the end, for every one of the 12 echolocation sound files, there existed a control sound file that contained essentially the same click sounds, occurring at the same temporal points, but devoid of any click echoes.

### Behavioral Testing Procedure for Angular Position Discrimination (EB and LB)

#### Active Echolocation

To determine angular position discrimination thresholds we employed a 2-Interval-2-AFC adaptive staircase method, with step-sizes in the first two trials computed based on [46], and in subsequent trials based on [47]. The participant's task on every trial was to actively echolocate and determine whether a position marker (described in main text) at a test position was located to the left or right of a position marker at a straight ahead reference position. Presentation was sequential. See Methods S1 for more details.

#### Passive Listening

During passive listening we used the same procedure as during active echolocation with the exception that participants did not actively echolocate, but listened to recordings of their own clicks and echoes. See Methods S1 for more details.

## Supporting Information

Figure S1 Results of source localization experiment. Plotted on the ordinate is the probability that the participant judges the source to be located to the right of its straight ahead reference position. Plotted on the abscissa is the position of the test position with respect to the straight ahead in degrees. Negative numbers indicate a position shift in the counter clockwise direction. Psychometric functions were obtained by fitting a 3-parameter sigmoid to the data. 25% and 75% thresholds and bias (denoted in red) were estimated from fitted curves. The zero-bias line is drawn for comparison (dashed line). It is evident from the data that EB and LB can determine the angular position of a source with high accuracy, i.e., thresholds for EB and LB are 2° and 2.5°, respectively. The localization thresholds for both EB and LB are within the range of what has been reported for source localization thresholds of sighted participants with respect to a centrally located reference source (Blauert, 1998; page 39, table 2.1). For both EB and LB, performance is slightly better during source localization than during active or passive echolocation (compare Figure 1 in main text). With regard to bias, the data show that EB is unbiased (red line at zero), but that LB tends to judge test locations to be to the left of the straight ahead (red line shifted to the right). This means, that LB's subjective straight ahead is shifted to the right. Thus, bias in source localization is similar to bias during active and passive echolocation for both participants (compare Figure 1 in main text).
(TIF)

Figure S2 BOLD activity projected on participants reconstructed and partially inflated cortical surface. Shown is the contrast between activations for outdoor recordings containing echoes from objects, and outdoor recordings that did not contain such echoes, evaluated at a more liberal statistical threshold then in the main text, i.e. p<.01 instead of p<.001 (compare Figure 3 in main text). Even at this more liberal statistical threshold, neither C1 nor C2 shows any difference in BOLD activity in visual cortex between echo and control conditions.
(TIF)

Figure S3 BOLD activity projected on participants reconstructed and partially inflated cortical surface. Marking of cortical surfaces and abbreviations as in Figure 2, main text. **Top panel:** BOLD activity in EB's and LB's brains while they listened to outdoor scene recordings (both echo and control sounds) and judged whether the recording contained echoes reflected from a car, tree or pole or no object echoes at all. Each participant listened to recordings of his own clicks and echoes as well as to recordings of the other person (see Figure 1G for behavioral results). EB shows highly reliable BOLD activity in the calcarine sulcus of the right hemisphere. LB shows activity at the apex of the occipital lobes of the right and left hemisphere, typically considered the ‘foveal part’ of visual cortex. Both participants also show BOLD activity in the lateral sulcus (i.e. Auditory Complex) of the left and right hemispheres, most likely due to the auditory nature of the stimuli. **Bottom panel:** BOLD activity in C1's and C2's brains while they listened to outdoor scene recordings (both echo and control sounds. The task was the same as for EB and LB, and each participant listened to recordings they had trained with as well as to the recordings of the other person, e.g. C1 listened to both EB's and LB's recordings (see Figure 1G for behavioral results). In contrast to EB and LB, neither C1 nor C2 show BOLD activity in calcarine sulcus. However, just as EB and LB, both C1 and C2 show robust BOLD activity in the lateral sulcus (i.e. Auditory Complex) of the left and right hemispheres.
(TIF)

Figure S4 BOLD activity in C1 and C2 brains that is related to recordings of echolocation sounds that convey movement to EB and LB, evaluated at a more liberal statistical threshold than reported in the main text, i.e. p<.01 instead of p<.001 (compare Figure 5 in main text). Also shown are areas sensitive to visual motion (area MT+) functionally defined at different significance levels (p<.05 (light green) or p<.05 Bonf. Corrected (dark green)). Even at this more liberal statistical threshold, neither C1 nor C2 show increased BOLD activity in regions posterior to the ITS/LOS junction for the contrast between ‘moving’ and ‘stationary’ echolocation stimuli.
(TIF)

Figure S5 BOLD activity in the cerebellum while participants listened to outdoor scene recordings (both echo and control sounds) and judged whether the recording contained echoes reflected from a car, tree or pole or no object echoes at all. Each EB and LB listened to recordings of his own clicks and echoes as well as to recordings of the other person. Similarly, each C1 and C2 listened to recordings he had trained with as well as to the recordings of the other person, e.g. C1 listened to both EB's and LB's recordings (see Figure 1G for behavioral results). Data are shown in neurological convention, i.e. left is left. Activity in the cerebellum was analyzed in stereotaxic space [49]. To evaluate significance of activity we used the same voxelwise significance thresholds as for cortical surface analyses for each participant. However, because the number of voxels in volume space differed from the number of vertices in surface space for each participant, the Bonferroni corrected significance level differs between cortex and cerebellum (compare Figure S3). To increase accuracy, cerebellar structures for each participant were identified based on anatomical landmarks. Structures were labeled according to the nomenclature developed by [26]. Data are not shown if no significant activity was found (empty cells in table).
(TIF)

Figure S6 Bird's eye view of the courtyard (highlighted in red) that was used to make outdoor scene recordings.
(TIF)

Figure S7 Illustrations of outdoor scenes used to make echolocation recordings (the participant stood in front of each object and made clicks) and background recordings used to make outdoor control sounds (the participant stood silently in front of each object).
(TIF)

Table S1 Expanded Classification Results (incl. sample size) for location, shape, motion and outdoor scenes experiments for EB and LB. Asterisks indicate that performance is significantly different from chance (p<.05). Unless otherwise indicated, chance performance is 50%. Tests of significance were only computed for entries in black (also contained in the main text). Sample sizes (shown in parenthesis) fulfill minimum requirement for confidence intervals for a proportion based on the normal approximation [48].
(DOC)

Table S2 Expanded Classification Results (incl. sample size) for location, shape, motion and outdoor scenes experiments for C1 and C2. Asterisks indicate that performance is significantly different from chance (p<.05). Unless otherwise indicated, chance performance is 50%. Tests of significance were only computed for entries in black (also contained in the main text). Sample sizes (shown in parenthesis) fulfill minimum requirement for confidence intervals for a proportion based on the normal approximation [48]. ^1^ = *less* than chance, because of bias to classify as ‘tree’.
(DOC)

Table S3 Statistical results of ROI analysis (contrast: Echo_Moving_−Echo_Stationary_ ) applied to area MT+ in C1 and C2. We applied regions of interest analysis to MT+ ROIs for both control participants to determine if the contrast Echo_Moving_−Echo_Stationary_ was significant (contrast values and SEM are shown in Figure 5, main text). It is evident that the contrast was not significant in any condition.
(DOC)

Table S4 Center-of-Gravity Talairach Coordinates for MT+ ROIs. For ROI selection methods see Methods S1.
(DOC)

Sound S1 Binaural recording of a click and click echoes made in EB's ears in the anechoic chamber, while he made a click in the presence of a position marker located 150 cm straight ahead. This sound accompanies Figure 1A, main text. NOTE: We advise to use in-ear stereo headphones to listen to sound sample.
(WAV)

Sound S2 Binaural recording of a click and click echoes made in LB's ears in the anechoic chamber, while he made a click in the presence of a position marker located 150 cm straight ahead. This sound accompanies Figure 1A, main text. NOTE: We advise to use in-ear stereo headphones to listen to sound sample.
(WAV)

Sound S3 Illustrations of sounds used during angular position discrimination – source localisation.Binaural recording of a click and click echoes made in SRA's ears in the anechoic chamber, while he listened to pseudo-clicks (derived from EB's original clicks) from a loudspeaker located 150 cm 10° to the right of straight ahead. In the experiment sounds were presented via MR compatible headphones (Sensimetrics, Malden, MA, USA, Model S-14). To illustrate the sounds that participants heard through these headphones during the experiments, sample sounds have been passed through a 10 kHz low-pass filter. NOTE: We advise to use in-ear stereo headphones to listen to sound sample.
(WAV)

Sound S4 Illustrations of sounds used during angular position discrimination – source localisation. Binaural recording of a click and click echoes made in SRA's ears in the anechoic chamber, while he listened to pseudo-clicks (derived from EB's original clicks) from a loudspeaker located 150 cm 10° to the left of straight ahead. In the experiment sounds were presented via MR compatible headphones (Sensimetrics, Malden, MA, USA, Model S-14). To illustrate the sounds that participants heard through these headphones during the experiments, sample sounds have been passed through a 10 kHz low-pass filter. NOTE: We advise to use in-ear stereo headphones to listen to sound sample.
(WAV)

Sound S5 Illustrations of sounds used during angular position discrimination – passive listening. Binaural recording of a click and click echoes made in EB's ears in the anechoic chamber, while he made clicks in the presence of a position marker located 150 cm 10° to the right of straight ahead. In the experiment sounds were presented via MR compatible headphones (Sensimetrics, Malden, MA, USA, Model S-14). To illustrate the sounds that participants heard through these headphones during the experiments, sample sounds have been passed through a 10 kHz low-pass filter. NOTE: We advise to use in-ear stereo headphones to listen to sound sample.
(WAV)

Sound S6 Illustrations of sounds used during angular position discrimination – passive listening. Binaural recording of a click and click echoes made in EB's ears in the anechoic chamber, while he made clicks in the presence of a position marker located 150 cm 10° to the left of straight ahead. In the experiment sounds were presented via MR compatible headphones (Sensimetrics, Malden, MA, USA, Model S-14). To illustrate the sounds that participants heard through these headphones during the experiments, sample sounds have been passed through a 10 kHz low-pass filter. NOTE: We advise to use in-ear stereo headphones to listen to sound sample.
(WAV)

Sound S7 Illustrations of sounds used during Shape/Location Classification. Binaural recording of click and click echoes made in LB's ears in the anechoic chamber, while he held his head stationary and made clicks in the presence of a concave surface located 40 cm and 20° to the left of straight ahead. In the experiment sounds were presented via MR compatible headphones (Sensimetrics, Malden, MA, USA, Model S-14). To illustrate the sounds that participants heard through these headphones during the experiments, sample sounds have been passed through a 10 kHz low-pass filter. NOTE: We advise to use in-ear stereo headphones to listen to sound sample.
(WAV)

Sound S8 Illustrations of sounds used during Shape/Location Classification. Binaural recording of click and click echoes made in LB's ears in the anechoic chamber, while he held his head stationary and made clicks in the presence of a flat surface located 40 cm and 20° to the left of straight ahead. In the experiment sounds were presented via MR compatible headphones (Sensimetrics, Malden, MA, USA, Model S-14). To illustrate the sounds that participants heard through these headphones during the experiments, sample sounds have been passed through a 10 kHz low-pass filter. NOTE: We advise to use in-ear stereo headphones to listen to sound sample.
(WAV)

Sound S9 Illustrations of sounds used during Motion Classification. Binaural recording of click and click echoes made in LB's ears in the anechoic chamber, while he moved his head randomly and made clicks in the presence of a concave surface located 40 cm and 20° to the left of straight ahead. In the experiment sounds were presented via MR compatible headphones (Sensimetrics, Malden, MA, USA, Model S-14). To illustrate the sounds that participants heard through these headphones during the experiments, sample sounds have been passed through a 10 kHz low-pass filter. NOTE: We advise to use in-ear stereo headphones to listen to sound sample.
(WAV)

Sound S10 Illustrations of sounds used during Motion Classification. Binaural recording of click and click echoes made in LB's ears in the anechoic chamber, while he moved his head in a sweeping motion from left to right and made clicks in the presence of a concave surface located 40 cm and 20° to the left of straight ahead. In th e experiment sounds were presented via MR compatible headphones (Sensimetrics, Malden, MA, USA, Model S-14). To illustrate the sounds that participants heard through these headphones during the experiments, sample sounds have been passed through a 10 kHz low-pass filter. NOTE: We advise to use in-ear stereo headphones to listen to sound sample.
(WAV)

Sound S11 Illustrations of sounds used during Motion Classification. Binaural recording of click and click echoes made in LB's ears in the anechoic chamber, while he held his head stationary and made clicks in the presence of a concave surface located 40 cm and 20° to the left of straight ahead. In the experiment sounds were presented via MR compatible headphones (Sensimetrics, Malden, MA, USA, Model S-14). To illustrate the sounds that participants heard through these headphones during the experiments, sample sounds have been passed through a 10 kHz low-pass filter. NOTE: We advise to use in-ear stereo headphones to listen to sound sample.
(WAV)

Sound S12 Illustrations of sounds used during Outdoor Scenes Classification. Binaural recording of clicks and click echoes made in EB's ears in an outdoor setting, while he made clicks in the presence of lamp-post located in front of him (background sounds contain birds, leaves, etc.). In the experiment sounds were presented via MR compatible headphones (Sensimetrics, Malden, MA, USA, Model S-14). To illustrate the sounds that participants heard through these headphones during the experiments, sample sounds have been passed through a 10 kHz low-pass filter. NOTE: We advise to use in-ear stereo headphones to listen to sound sample.
(WAV)

Sound S13 Illustrations of sounds used during Outdoor Scenes Classification. Control sound for Sound S12. This sound contains background sounds very similar to those in Sound S12, as the recording was also made in EB's ears in an outdoor setting while he stood in front of the lamp post. However, during the recording EB was silent. The click-like sounds in the audio file are Pseudo-clicks derived from EB's own clicks but placed in the same positions as the original clicks in Sound S12 (see Methods S1). Thus, the control sound is yoked to the Sound 12, but does not contain click-echoes. In the experiment sounds were presented via MR compatible headphones (Sensimetrics, Malden, MA, USA, Model S-14). To illustrate the sounds that participants heard through these headphones during the experiments, sample sounds have been passed through a 10 kHz low-pass filter. NOTE: We advise to use in-ear stereo headphones to listen to sound sample.
(WAV)

Audiology Report S1 Summary of audiological test results for EB and LB (Air Conduction Thresholds, Tympanograms, Acoustic Reflex Thresholds, Distortion Product Otoacoustic Emissions).
(PDF)

Methods S1 Additional information about the experimental methods.
(DOC)
