# Supplementary figures and images for: Neural Correlates of Natural Human Echolocation in Early and Late Blind Echolocation Experts
Source: PLoS One. 2011 May 25;6(5):e20162. doi: 10.1371/journal.pone.0020162 (PMC3102086; doi:10.1371/journal.pone.0020162)

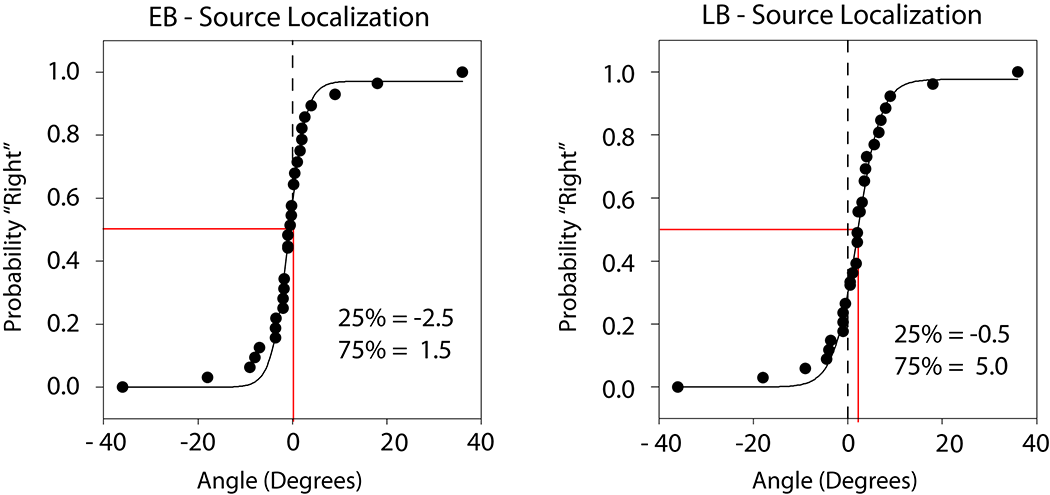

Supplement: Figure S1 — Results of source localization experiment. Plotted on the ordinate is the probability that the participant judges the source to be located to the right of its straight ahead reference position. Plotted on the abscissa is the position of the test position with respect to the straight ahead in degrees. Negative numbers indicate a position shift in the counter clockwise direction. Psychometric functions were obtained by fitting a 3-parameter sigmoid to the data. 25% and 75% thresholds and bias (denoted in red) were estimated from fitted curves. The zero-bias line is drawn for comparison (dashed line). It is evident from the data that EB and LB can determine the angular position of a source with high accuracy, i.e., thresholds for EB and LB are 2° and 2.5°, respectively. The localization thresholds for both EB and LB are within the range of what has been reported for source localization thresholds of sighted participants with respect to a centrally located reference source (Blauert, 1998; page 39, table 2.1). For both EB and LB, performance is slightly better during source localization than during active or passive echolocation (compare Figure 1 in main text). With regard to bias, the data show that EB is unbiased (red line at zero), but that LB tends to judge test locations to be to the left of the straight ahead (red line shifted to the right). This means, that LB's subjective straight ahead is shifted to the right. Thus, bias in source localization is similar to bias during active and passive echolocation for both participants (compare Figure 1 in main text). (TIF) [file pone.0020162.s001.tif]

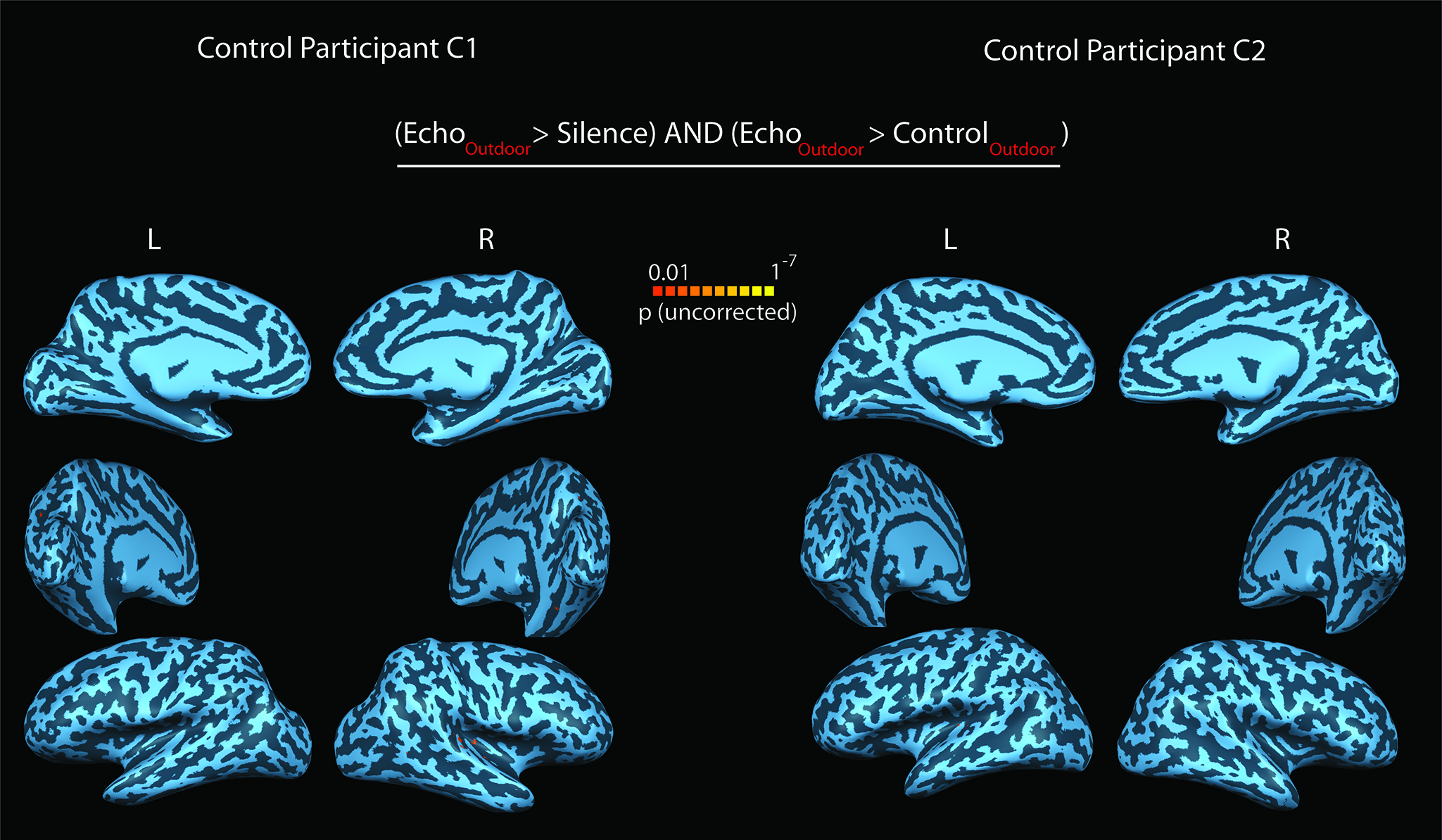

Supplement: Figure S2 — BOLD activity projected on participants reconstructed and partially inflated cortical surface. Shown is the contrast between activations for outdoor recordings containing echoes from objects, and outdoor recordings that did not contain such echoes, evaluated at a more liberal statistical threshold then in the main text, i.e. p<.01 instead of p<.001 (compare Figure 3 in main text). Even at this more liberal statistical threshold, neither C1 nor C2 shows any difference in BOLD activity in visual cortex between echo and control conditions. (TIF) [file pone.0020162.s002.tif]

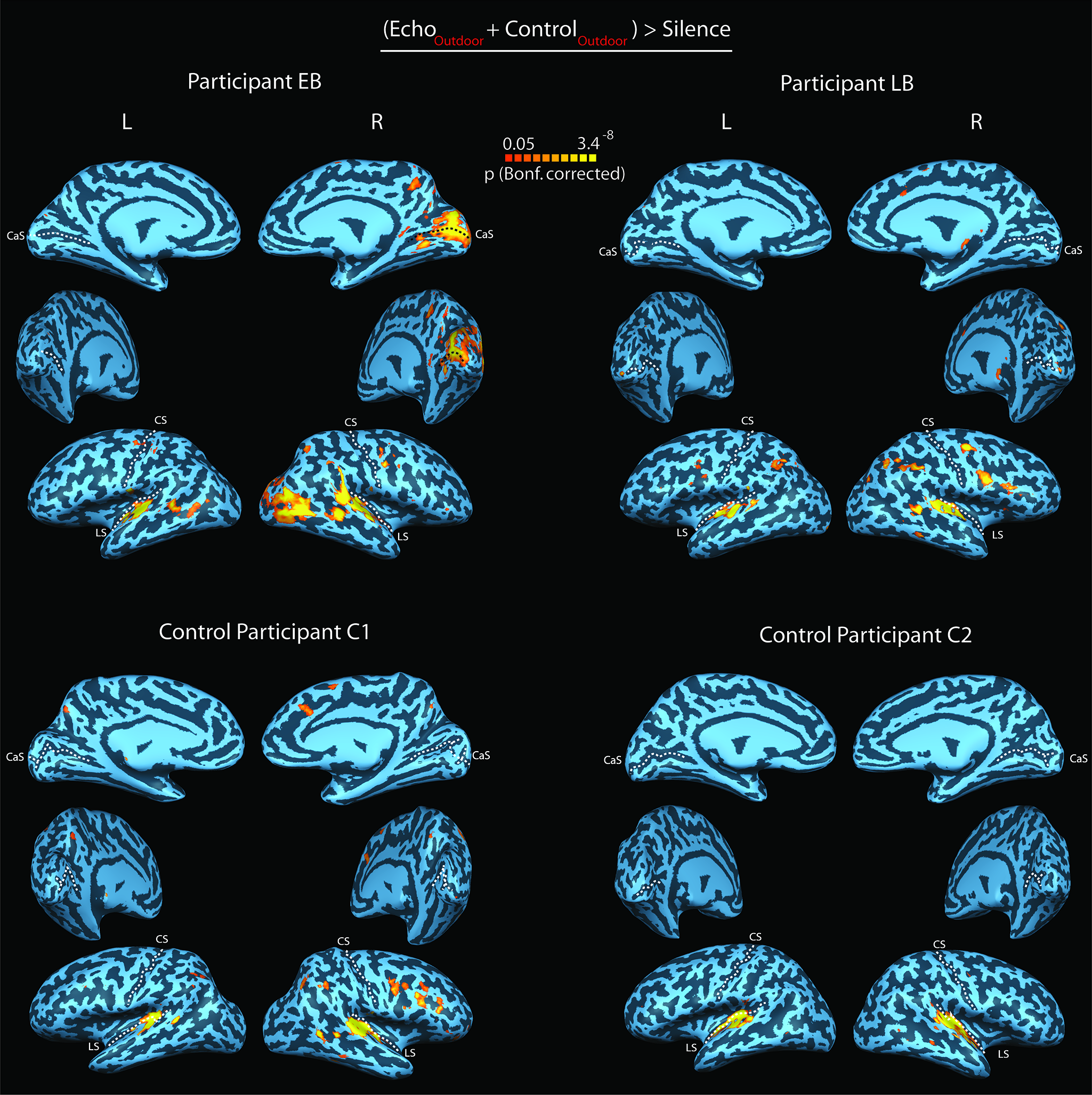

Supplement: Figure S3 — BOLD activity projected on participants reconstructed and partially inflated cortical surface. Marking of cortical surfaces and abbreviations as in Figure 2, main text. Top panel: BOLD activity in EB's and LB's brains while they listened to outdoor scene recordings (both echo and control sounds) and judged whether the recording contained echoes reflected from a car, tree or pole or no object echoes at all. Each participant listened to recordings of his own clicks and echoes as well as to recordings of the other person (see Figure 1G for behavioral results). EB shows highly reliable BOLD activity in the calcarine sulcus of the right hemisphere. LB shows activity at the apex of the occipital lobes of the right and left hemisphere, typically considered the ‘foveal part’ of visual cortex. Both participants also show BOLD activity in the lateral sulcus (i.e. Auditory Complex) of the left and right hemispheres, most likely due to the auditory nature of the stimuli. Bottom panel: BOLD activity in C1's and C2's brains while they listened to outdoor scene recordings (both echo and control sounds. The task was the same as for EB and LB, and each participant listened to recordings they had trained with as well as to the recordings of the other person, e.g. C1 listened to both EB's and LB's recordings (see Figure 1G for behavioral results). In contrast to EB and LB, neither C1 nor C2 show BOLD activity in calcarine sulcus. However, just as EB and LB, both C1 and C2 show robust BOLD activity in the lateral sulcus (i.e. Auditory Complex) of the left and right hemispheres. (TIF) [file pone.0020162.s003.tif]

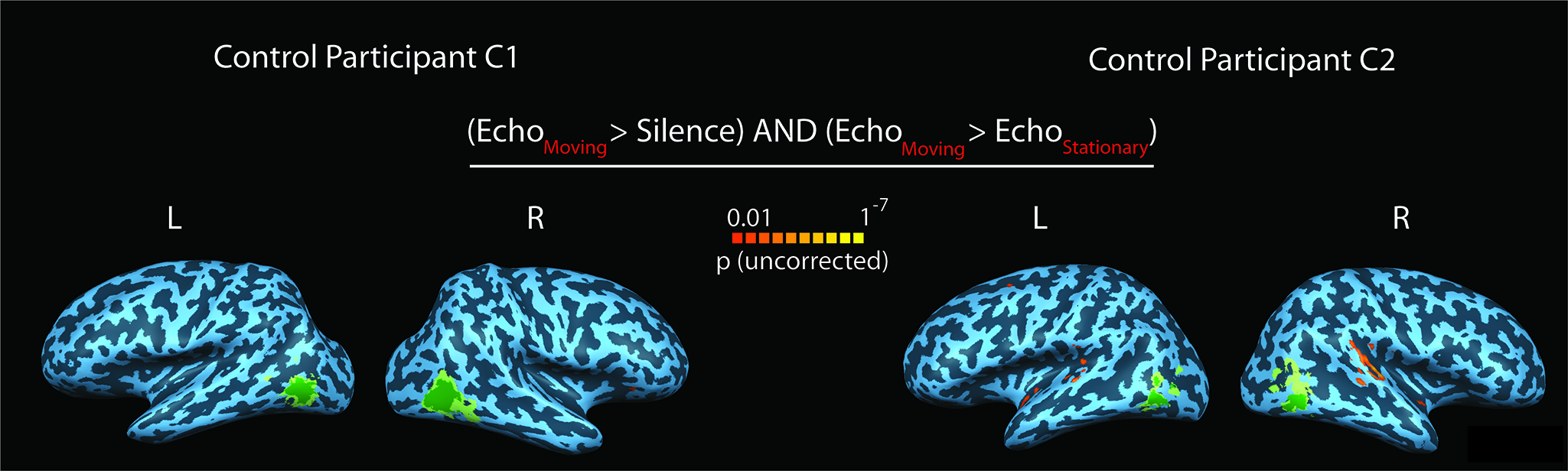

Supplement: Figure S4 — BOLD activity in C1 and C2 brains that is related to recordings of echolocation sounds that convey movement to EB and LB, evaluated at a more liberal statistical threshold than reported in the main text, i.e. p<.01 instead of p<.001 (compare Figure 5 in main text). Also shown are areas sensitive to visual motion (area MT+) functionally defined at different significance levels (p<.05 (light green) or p<.05 Bonf. Corrected (dark green)). Even at this more liberal statistical threshold, neither C1 nor C2 show increased BOLD activity in regions posterior to the ITS/LOS junction for the contrast between ‘moving’ and ‘stationary’ echolocation stimuli. (TIF) [file pone.0020162.s004.tif]

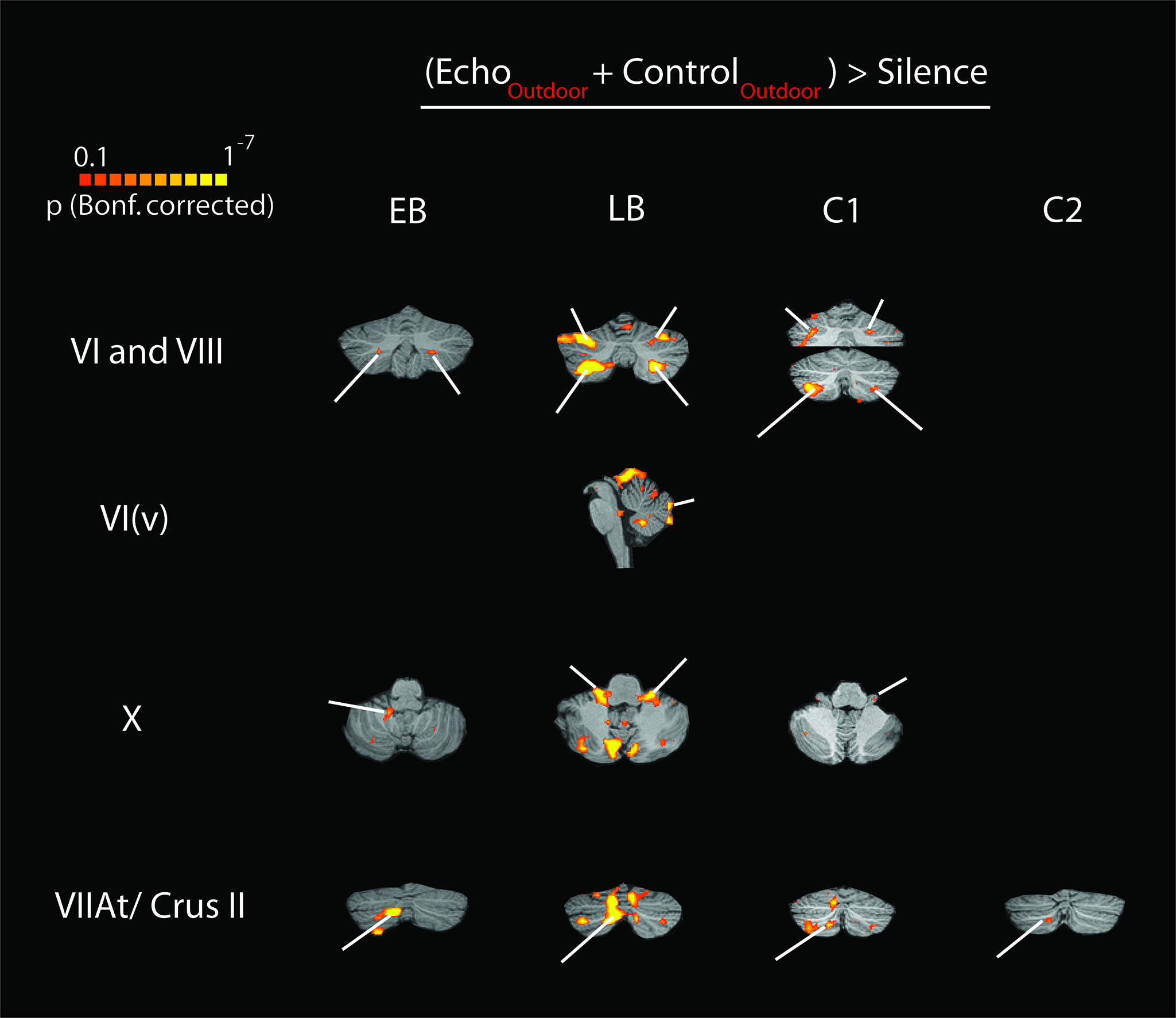

Supplement: Figure S5 — BOLD activity in the cerebellum while participants listened to outdoor scene recordings (both echo and control sounds) and judged whether the recording contained echoes reflected from a car, tree or pole or no object echoes at all. Each EB and LB listened to recordings of his own clicks and echoes as well as to recordings of the other person. Similarly, each C1 and C2 listened to recordings he had trained with as well as to the recordings of the other person, e.g. C1 listened to both EB's and LB's recordings (see Figure 1G for behavioral results). Data are shown in neurological convention, i.e. left is left. Activity in the cerebellum was analyzed in stereotaxic space [49]. To evaluate significance of activity we used the same voxelwise significance thresholds as for cortical surface analyses for each participant. However, because the number of voxels in volume space differed from the number of vertices in surface space for each participant, the Bonferroni corrected significance level differs between cortex and cerebellum (compare Figure S3). To increase accuracy, cerebellar structures for each participant were identified based on anatomical landmarks. Structures were labeled according to the nomenclature developed by [26]. Data are not shown if no significant activity was found (empty cells in table). (TIF) [file pone.0020162.s005.tif]

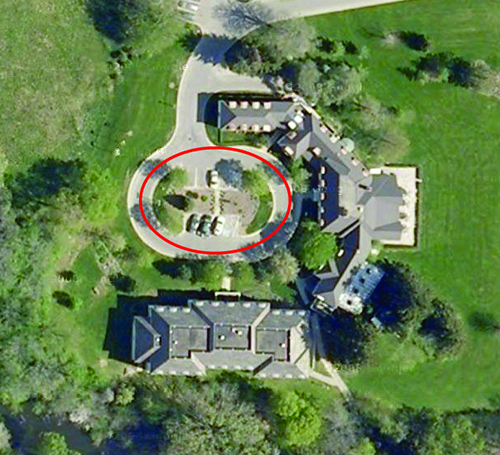

Supplement: Figure S6 — Bird's eye view of the courtyard (highlighted in red) that was used to make outdoor scene recordings. (TIF) [file pone.0020162.s006.tif]

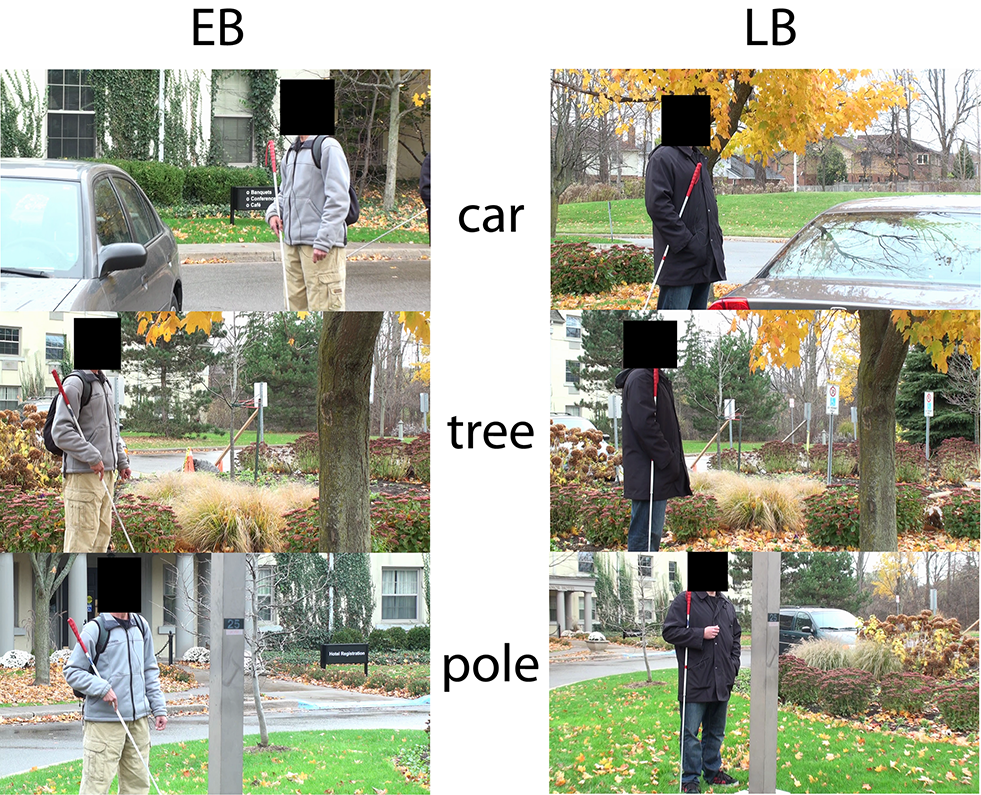

Supplement: Figure S7 — Illustrations of outdoor scenes used to make echolocation recordings (the participant stood in front of each object and made clicks) and background recordings used to make outdoor control sounds (the participant stood silently in front of each object). (TIF) [file pone.0020162.s007.tif]
