# Supplementary material for: Neural Correlates of Natural Human Echolocation in Early and Late Blind Echolocation Experts
Source: PLoS One. 2011 May 25;6(5):e20162. doi: 10.1371/journal.pone.0020162 (PMC3102086; doi:10.1371/journal.pone.0020162)
Supplement: Table S1 — Expanded Classification Results (incl. sample size) for location, shape, motion and outdoor scenes experiments for EB and LB. Asterisks indicate that performance is significantly different from chance (p<.05). Unless otherwise indicated, chance performance is 50%. Tests of significance were only computed for entries in black (also contained in the main text). Sample sizes (shown in parenthesis) fulfill minimum requirement for confidence intervals for a proportion based on the normal approximation [48]. (DOC) [file pone.0020162.s008.doc]

**Table S1-** Expanded Classification Results (incl. sample size) for location, shape, motion and outdoor scenes experiments for EB and LB. Asterisks indicate that performance is significantly different from chance (p<.05). Unless otherwise indicated, chance performance is 50%. Tests of significance were only computed for entries in black (also contained in the main text). Sample sizes (shown in parenthesis) fulfill minimum requirement for confidence intervals for a proportion based on the normal approximation [48].

| **Location Classification (left/right)** | **EB**  (n = 32) | **LB**  (n = 32) |
| --- | --- | --- |
| % correct (all) | 100% (32/32)* | 50% (16/32) |
| % correct (right) | 100% (16/16) | 43.75% (7/16) |
| % correct (left) | 100% (16/16) | 56.25% (9/16) |
| % ‘left’ | 50% (32/32) | 56.25% (18/32) |
| **Shape Classification (concave/flat**) | **EB**  (n=32) | **LB**  (n=32) |
| % correct (all) | 75% (24/32)* | 65.625% (21/32)* |
| % correct (concave) | 75% (12/16) | 56.25% (9/16) |
| % correct (flat) | 75% (12/16) | 75% (12/16) |
| % ‘flat’ | 50%­ (16/32) | 59.375% (19/32) |
| **Motion Classification**  **(stationary/moving)** | **EB**  (n=64) | **LB**  (n=64) |
| % correct (all) | 100% (64/64)* | 76.56% (49/64)* |
| % correct (stationary) | 100% (32/32) | 84.38% (27/32) |
| % correct (moving) | 100% (32/32) | 68.75% (22/32) |
| % correct (as sweep) | 100% (16/16) | 43.75% (7/16) |
| % correct (as random) | 100% (16/16) | 93.75% (15/16) |
| % ‘stationary’ | 50% (64/64) | 57.8% (37/64) |
| **Scene Classification**  **(car/pole/tree/nothing)** | **EB**  (n=96) | **LB**  (n=94) |
| % correct (something vs. nothing) | 100% (96/96)* | 96.8% (91/94)* |
| % correct (as EB) | 100% (48/48)* | 95.7% (45/47)* |
| % correct (as LB) | 100% (48/48)* | 97.9% (46/47)* |
| % correct (chance = 41.7%)  (car vs. pole vs. tree vs. nothing) | 99% (95/96)* | 81.9% (77/94)* |
| % correct (as EB) | 97.9% (47/48)* | 85.1%(40/47)* |
| % correct (as LB) | 100% (48/48)* | 78.7%(37/47)* |
