# Supplementary material for: Neural Correlates of Natural Human Echolocation in Early and Late Blind Echolocation Experts
Source: PLoS One. 2011 May 25;6(5):e20162. doi: 10.1371/journal.pone.0020162 (PMC3102086; doi:10.1371/journal.pone.0020162)
Supplement: Table S2 — Expanded Classification Results (incl. sample size) for location, shape, motion and outdoor scenes experiments for C1 and C2. Asterisks indicate that performance is significantly different from chance (p<.05). Unless otherwise indicated, chance performance is 50%. Tests of significance were only computed for entries in black (also contained in the main text). Sample sizes (shown in parenthesis) fulfill minimum requirement for confidence intervals for a proportion based on the normal approximation [48]. 1 = less than chance, because of bias to classify as ‘tree’. (DOC) [file pone.0020162.s009.doc]

Table S2- Expanded Classification Results (incl. sample size) for location, shape, motion and outdoor scenes experiments for C1 and C2. Asterisks indicate that performance is significantly different from chance (p<.05). Unless otherwise indicated, chance performance is 50%. Tests of significance were only computed for entries in black (also contained in the main text). Sample sizes (shown in parenthesis) fulfill minimum requirement for confidence intervals for a proportion based on the normal approximation [48]. ^1^ = *less* than chance, because of bias to classify as ‘tree’

| **Location Classification (left/right)** | **C1**  (n=27) | **C2**  (n=40) |
| --- | --- | --- |
| % correct (all) | 44.44 % (12/27) | 50% (20/40) |
| % correct (right) | 47.06% (8/17) | 40% (8/20) |
| % correct (left) | 40% (4/10) | 60% (12/20) |
| % ‘left’ | 48.15% (13/27) | 60% (24/40) |
| **Shape Classification (concave/flat**) | **C1**  (n=29) | **C2**  (n=40) |
| % correct (all) | 48.28% (14/29) | 45% (18/40) |
| % correct (concave) | 38.46% (5/13) | 50% (10/20) |
| % correct (flat) | 56.25% (9/16) | 40% (8/20) |
| % ‘flat’ | 58.62%­ (17/29) | 45% (18/40) |
| **Motion Classification**  **(stationary/moving)** | **C1**  (n=78) | **C2**  (n=80) |
| % correct (all) | 67.95% (53/78)* | 95% (76/80)* |
| % correct (stationary) | 82.05% (32/39) | 97.5% (39/40) |
| % correct (moving) | 53.85% (21/39) | 92.5% (37/40) |
| % correct (as sweep) | 70% (14/20) | 100% (20/20) |
| % correct (as random) | 36.84% (7/19) | 85% (17/20) |
| % ‘stationary’ | 64.1% (50/78) | 52.5% (42/80) |
| **Scene Classification**  **(car/pole/tree/nothing)** | **C1**  (n=64) | **C2**  (n=96) |
| % correct (something vs. nothing) | 71.9% (46/64)* | 66.7% (64/96)* |
| % correct (as EB) | 78.1% (25/32)* | 58.3% (28/48) |
| % correct (as LB) | 65.6% (21/32)* | 75% (36/48)* |
| % correct (chance = 41.7%)  (car vs. pole vs. tree vs. nothing) | 37.5% (24/64) | 36.5% (35/96) |
| % correct (as EB) | 43.8% 14/32) | 20.8% (10/48)*^1^ |
| % correct (as LB) | 34.4%(11/32) | 52.1% (25/48) |
