# Supplementary material for: Neural Correlates of Natural Human Echolocation in Early and Late Blind Echolocation Experts
Source: PLoS One. 2011 May 25;6(5):e20162. doi: 10.1371/journal.pone.0020162 (PMC3102086; doi:10.1371/journal.pone.0020162)
Supplement: Table S3 — Statistical results of ROI analysis (contrast: EchoMoving−EchoStationary ) applied to area MT+ in C1 and C2. We applied regions of interest analysis to MT+ ROIs for both control participants to determine if the contrast EchoMoving−EchoStationary was significant (contrast values and SEM are shown in Figure 5, main text). It is evident that the contrast was not significant in any condition. (DOC) [file pone.0020162.s010.doc]

Table S3 - Statistical results of ROI analysis (contrast: Echo_Moving_ – Echo_Stationary_ ) applied to area MT+ in C1 and C2. We applied regions of interest analysis to MT+ ROIs for both control participants to determine if the contrast Echo_Moving_ – Echo_Stationary_ was significant (contrast values and SEM are shown in Figure 5, main text). It is evident that the contrast was not significant in any condition.

|  |  | **Statistical result** |
| --- | --- | --- |
|  |  |  |
| **C1** | **P<.05** | t(118)= -.75; p=0.4549 |
|  | **P<.05 (Bonf.corr.)** | t(118)= -.725; p=0.4698 |
|  |  |  |
| **C2** | **P<.05** | t(113)= -.465; p=0.6425 |
|  | **P<.05 (Bonf.corr.)** | t(113)= -.731; p=0.4663 |
