# Supplementary material for: Neural Correlates of Natural Human Echolocation in Early and Late Blind Echolocation Experts
Source: PLoS One. 2011 May 25;6(5):e20162. doi: 10.1371/journal.pone.0020162 (PMC3102086; doi:10.1371/journal.pone.0020162)
Supplement: Table S4 — Center-of-Gravity Talairach Coordinates for MT+ ROIs. For ROI selection methods see Methods S1. (DOC) [file pone.0020162.s011.doc]

Table S4 - Center-of-Gravity Talairach Coordinates for MT+ ROIs. For ROI selection methods see Supplementary Methods S1.

|  |  | **LH** |  |  |  | **RH** |  |  |
| --- | --- | --- | --- | --- | --- | --- | --- | --- |
|  |  | **x** | **y** | **z** |  | **x** | **y** | **z** |
| **C1** | **P<.05** | -47 | -68 | 2.6 |  | 42 | -59 | -3.6 |
|  | **P<.05 (Bonf.corr.)** | -48 | -69 | 3.3 |  | 38 | -61 | -1.7 |
|  |  |  |  |  |  |  |  |  |
| **C2** | **P<.05** | -45 | -69 | 1.4 |  | 43 | -63 | 3.4 |
|  | **P<.05 (Bonf.corr.)** | -45 | -70 | 0.58 |  | 43 | -62 | -1.7 |
