# Supplementary material for: Neural Correlates of Natural Human Echolocation in Early and Late Blind Echolocation Experts
Source: PLoS One. 2011 May 25;6(5):e20162. doi: 10.1371/journal.pone.0020162 (PMC3102086; doi:10.1371/journal.pone.0020162)
Supplement: Audiology Report S1 — Summary of audiological test results for EB and LB (Air Conduction Thresholds, Tympanograms, Acoustic Reflex Thresholds, Distortion Product Otoacoustic Emissions). (PDF) [file pone.0020162.s025.pdf]

Audiology Report EB

Air Conduction Thresholds (**Madsen ITERA**)

Frequency (Hz)	250	500	750	1000	1500	2000	3000	4000	6000
Left (dB HL)	0	10	0	0	0	0	0	0	0
Right (dB HL)	0	10	15	10	5	0	0	0	5

Setup: To set-up for this part of the test, the participant is asked to wear a set of TDH headphones and is instructed to respond to tones presented to them one ear at a time by pressing a button. The audiometer, a device consisting of a stimulus generator and an attenuator, is used to vary both the frequency and the level of the tone heard by the participant. The goal of this part of the study is to determine the lowest level the participant responds to the tone and as such, to determine the threshold of hearing for the individual at each frequency. The tones are presented in accordance with the Hughson-Westlake procedure [1].

Comments: The term air conduction threshold is used because the behavioural threshold of hearing is determined through the use of THD headphones. The tones produced by the audiometer are transmitted through the air from these headphones, through the external auditory canal, across the middle ear and to the inner ear. Air conduction thresholds for a normal hearing individual are characterized as being below 25 dB HL [1]. As such, this patient has normal thresholds at all frequencies for both their right and left ears.

The participant commented on the fact that he felt he could hear better from his left ear. An otoscopic examination revealed that his left ear canal had a fairly large bend and was much narrower than his right. Also, the above air conduction thresholds support the patient's observation of his own hearing ability. At 750Hz and 1000Hz, the left ear has a significantly better hearing threshold than the right. This amount of asymmetry between ears is normal.

Tympanometry (**Madsen OTOflex100**)

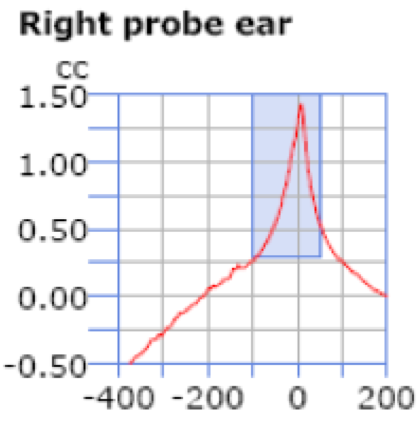

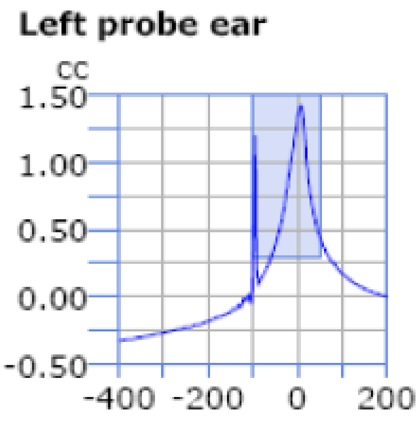

Measurement	Right Ear	Left Ear
Tympanic Peak Pressure (daPa)	6	5
Static Admittance (mL)	1.41	1.41
Tympanic Width (daPa)	72	60
Acoustic Reflex Threshold, CONTRA, BBN (dB)	75	85

Setup: To set up for this part of the test, two inserts were put into the participants ears; a probe placed in the ear being tested and a foam insert placed into the other ear. The probe consists of a sound transducer, a microphone and a pressure pump. The foam insert is simply a sound transducer.

Tympanometry: The probe tip delivers a constant probe tone at 226 Hz while adjusting the pressure in the ear canal from +200 daPa to -200 daPa. By adjusting the pressure in the ear canal and measuring the amount of the 226 Hz probe tone that is reflected back to the microphone of the probe, a measure is made of the static admittance of the system. The admittance of the system increases as the pressure in the middle ear and the pressure in the ear canal become equivalent.

The static admittance value of the system is determined by subtracting the admittance value at +200 daPa from the admittance value at peak pressure. In a normally functioning middle ear system, the peak pressure will fall between +50 and -150 daPa [2]. As such, the peak pressures for this patient would indicate normal middle ear functioning. The 90th percentile normal static admittance range is between 0.4 and 1.5 mL [2]. The results above fall a bit above this normal range but would still be considered to be normal.

The tympanic width is often used to help diagnose certain diseases of the middle ear. The 90th percentile normal range for tympanic width is 60 – 150 daPa [2]. Both the left and right ears of this participant would be considered healthy and normal (free of disease).

The shape of a normal tympanogram is triangular with the peak of the triangle corresponding to the maximum compliance of the system. An example of this ideal tympanogram shape can be seen for the right ear of this participant. The left ear, however, has an added narrow peak at around -100 daPa. This peak is usually characteristic of a pinhole perforation of the tympanic membrane. A pinhole perforation is very small and shouldn't have an adverse effect on day to day activities. Patients should be careful not to get a significant amount of water into the ear canal as it may travel through this hole into the middle ear cavity. This perforation could have resulted from a recent flight. Common protocol in this case is to refer on to an Audiologist to take a closer look. Generally, these pinhole perforations will heal on their own.

Reflex Threshold Testing: To test the middle ear muscle reflex threshold of this participant, a broadband stimulus was presented to the ear opposite the probe. In humans, the middle ear muscle reflex is a contraction of the stapedius muscle in the middle ear. This contraction changes the transmission of sound to the cochlea and can be used as a measure of the integrity of this auditory neural pathways in the brain stem. The intensity of this broadband stimulus was changed until the contralateral acoustic reflex was activated (i.e. if the broadband stimuli was delivered to the left ear, the broadband stimuli would be increased in level until the reflex in the right ear was activated). The reflex activation is measured by the stiffening of the ear drum and corresponding decrease in admittance. The broadband stimulus is increased in 10 dB increments until a change in admittance of at least 0.02 mL is measured. Normal broadband acoustic reflex thresholds generally fall between 60 and 95 dB HL [2]. Thus, we can reasonably conclude that the acoustic reflex thresholds for this participant are normal for both the left and right ears.

Distortion Product Otoacoustic Emissions (**Custom Software with Etymotic ER10B + probe**)

A distortion product otoacoustic emission is an acoustic emission produced by the cochlea when two stimulus tones, labelled f1 and f2, are presented to the ear. The distortion products created by the cochlea are related to outer hair cell (OHC) function within the cochlea. OHC's, which are located along the basilar membrane, contribute to the frequency selectivity, the wide dynamic range, and the overall sensitivity of the cochlea [3]. Each tone has a characteristic place along the basilar membrane at which maximal motion will occur. The amount of motion at each tone's characteristic place is related to the level of the tone presented to the ear. The DPOAE is produced at the overlap region of the two tones on the basilar membrane. To optimize the measured DPOAE, stimulus levels were set to 65 dB SPL for f1 and 55 dB SPL for f2, with a f2/f1 ratio of 1.22 [3]. We have tested both the participants left and right ears at five f2 frequencies; 1000, 1500, 2000, 3000 and 4000 Hz. We then compared the participant's emission level to established norms [3] and those obtained from young normal hearing individuals in our laboratory.

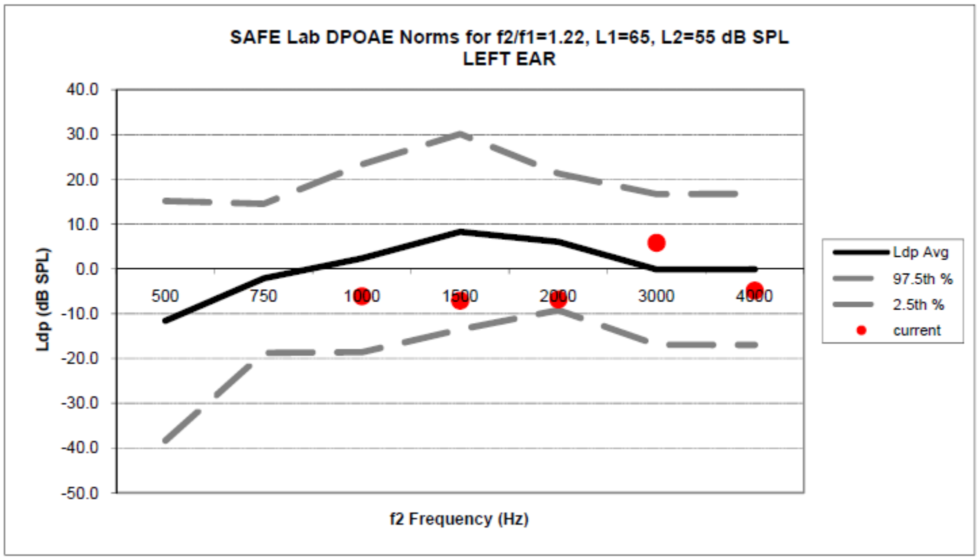

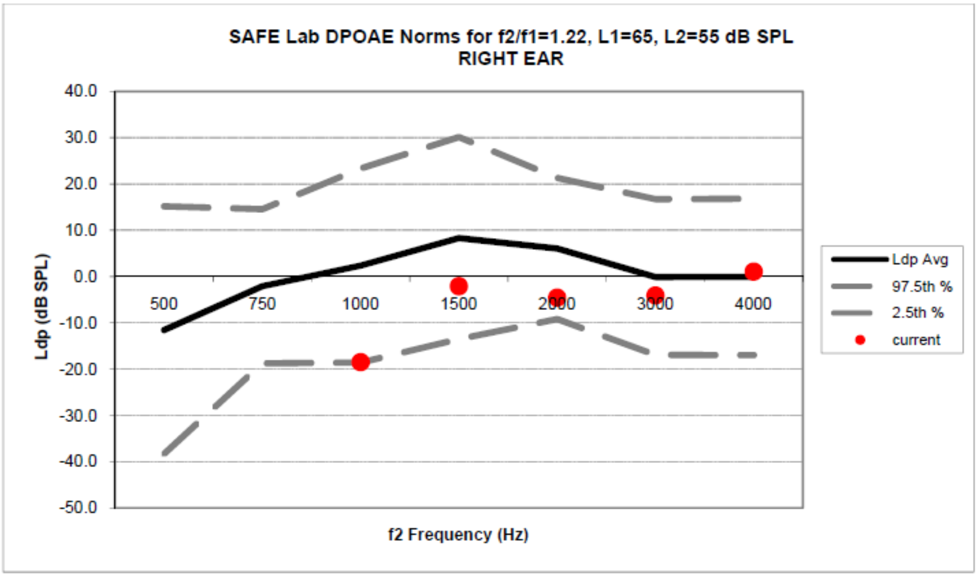

Interpretation: The upper grey dashed line shows the 97.5th percentile for DPOAE's produced in normal hearing ears in our laboratory. The lower grey dashed line gives the 2.5th percentile for DPOAE's produced by normal hearing ears. The middle black line shows the average DPOAE level found in normal hearing ears. The measurements made for this participant are shown with red dots. This participant's DPOAE levels fall within normal expectations. Therefore, we have not observed any evidence of OHC dysfunction, however some normal hearing ears do produce larger emissions.

Audiology Report LB

Air Conduction Thresholds (**Madsen ITERA**)

Frequency (Hz)	250	500	750	1000	1500	2000	3000	4000	6000
Left HL	*0*	*5*	*0*	*0*	*0*	*0*	*5*	*-5*	*0*
Right HL	*5*	*5*	*0*	*0*	*0*	*-5*	*5*	*0*	*10*

Comments: Air conduction thresholds for a normal hearing individual are characterized as being below 25 dB HL. As such, this patient has normal thresholds at all frequencies for both the right and left ears. Otoscopy revealed that this participant had some wax build-up in the ear canal of both ears.

Tympanometry (**Madsen OTOflex100**)

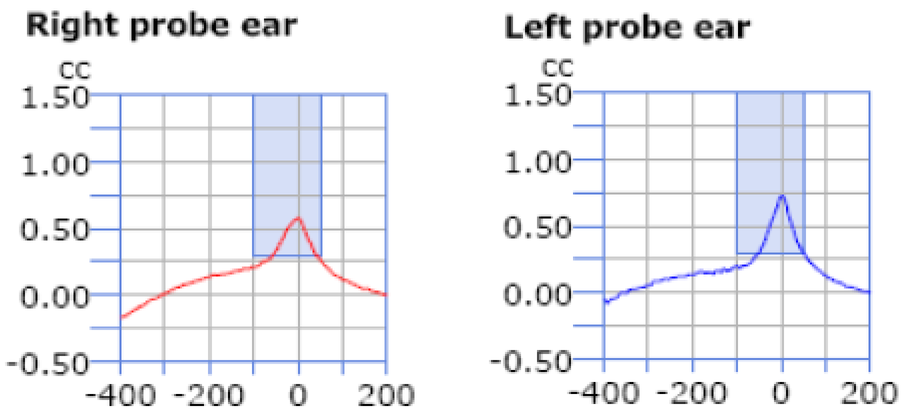

Measurement	Right Ear	Left Ear
Typanic Peak Pressure (daPa)	-2	1
Static Admittance (mL)	0.58	0.73
Tympanic Width (daPa)	103	80
Acoustic Reflex Threshold, CONTRA, BBN (dB)	d.n.e	70

Tympanometry:

In a normally functioning middle ear system, the peak pressure will fall between +50 and -150 daPa. As such, the peak pressures for this participant would indicate normal middle ear functioning. The 90th percentile normal static admittance range is between 0.4 and 1.5 mL [2]. The results above fall into this 90th percentile normal range for both the right and left ears.

The tympanic width is often used to help diagnose certain diseases of the middle ear. The 90th percentile normal range for tympanic width is 50 – 110 daPa [2]. Both the left and right ears of this participant would be considered healthy and normal (free of disease).

The shape of a normal tympanogram is triangular with the peak of the triangle corresponding to the maximum compliance of the system. Examples of this ideal tympanogram shape can be seen for both the right and left ears of this participant.

Reflex Threshold Testing: Normal broadband acoustic reflex thresholds generally fall between 60 and 95 dB HL. For this particular test, we artificially set the maximum intensity of the broadband noise to 85 dB HL, as to avoid presenting loud sounds to our guests (in case they were sensitive to loud sounds). As such, we can reasonably conclude that this participant had an acoustic reflex within the normal range for the left ear, but due to the limit set on the equipment, we do not have enough information to make a conclusion for the right ear.

<u>Distortion Product Otoacoustic Emissions (**Custom Software with Etymotic ER10B + probe**)</u>

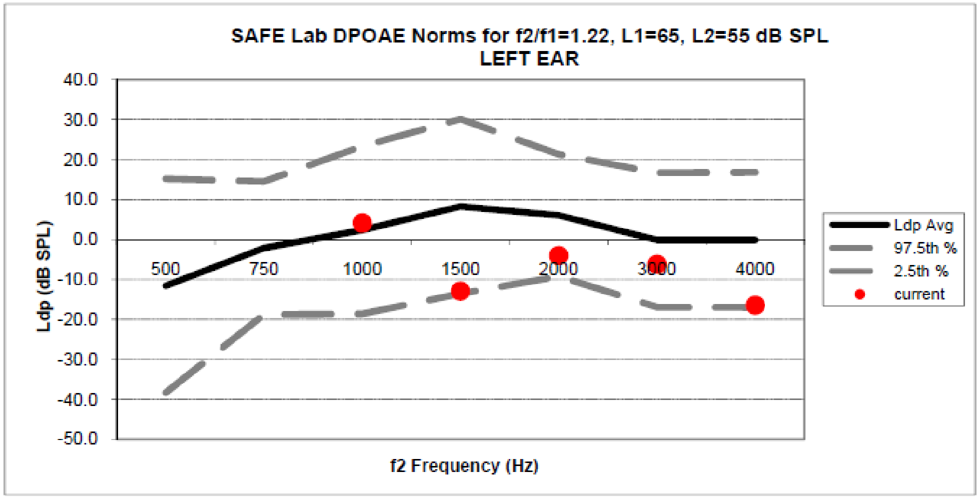

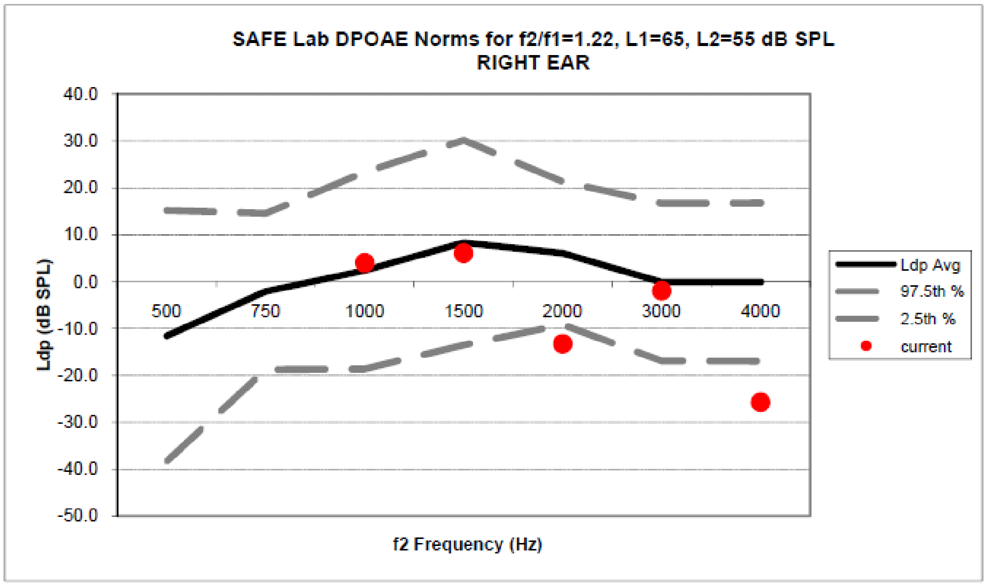

Interpretation: The upper grey dashed line shows the 97.5th percentile for DPOAE's produced in normal hearing ears. The lower grey dashed line gives the 2.5th percentile for DPOAE's produced by normal hearing ears. The middle black line shows the average DPOAE level found in normal hearing ears. The measurements made for this participant are shown with red dots. DPOAEs were statistically larger than the background noise across all frequencies in both ears and the DPOAE amplitudes in the left ear have fallen within the normal limits. In the right ear, however, the DPOAE's at 2000Hz and 4000Hz have fallen

below the normal limits. This may be in part due to the wax in this individual's ear canal. Due to the fact that valid DPOAE measurements were recorded across all frequencies, we have not observed any evidence of OHC dysfunction, however some normal hearing ears do produce larger emissions.

REFERENCES:

1. Roeser RJ, Clark JL (2007) Pure Tone Tests. In: Roeser RJ, Valente M, Hosford-Dunn H, editors. Audiology: Diagnosis, 2nd ed. New York: Thieme Medical Publishers. pp.238 – 260.
2. Clark JL, Roeser RJ, Mendrygal M (2007) Middle Ear Measures. In: Roeser RJ, Valente M, Hosford-Dunn H, editors. Audiology: Diagnosis, 2nd ed. New York: Thieme Medical Publishers. pp. 380-399.
3. Gorga MP, Neely ST, Ohlrich B, Hoover B, Redner J, Peters J (1997) From Laboratory to Clinic: A Large Scale Study of Distortion Product Otoaccoustic Emissions in Ears with Normal Hearing and Ears with Hearing Loss. J Acoust Soc Am 18*:* 440-455.
