# Supplementary material for: Neural Correlates of Natural Human Echolocation in Early and Late Blind Echolocation Experts
Source: PLoS One. 2011 May 25;6(5):e20162. doi: 10.1371/journal.pone.0020162 (PMC3102086; doi:10.1371/journal.pone.0020162)
Supplement: Methods S1 — Additional information about the experimental methods. (DOC) [file pone.0020162.s026.doc]

SUPPLEMENTARY METHODS S1

[1. Participants EB and LB – Case Histories 2](#__RefHeading___Toc286401431)

[2. Binaural Microphone Calibration 2](#__RefHeading___Toc286401432)

[3. Sound Equalization 2](#__RefHeading___Toc286401433)

[4. Description of Echolocation Clicks 2](#__RefHeading___Toc286401434)

[5. Experimental Stimuli - Spatial Source Localization 3](#__RefHeading___Toc286401435)

[5.1. Setup and General Recording Procedure (Anechoic Chamber) 3](#__RefHeading___Toc286401436)

[5.2. Experimental Stimuli and Sound Editing 3](#__RefHeading___Toc286401437)

[6. Behavioral Testing Procedures (EB and LB) 4](#__RefHeading___Toc286401438)

[6.1. Prior to fMRI 4](#__RefHeading___Toc286401439)

[6.2. During fMRI (see main text: fMRI functional and behavioral paradigm) 6](#__RefHeading___Toc286401440)

[6.3. After fMRI 6](#__RefHeading___Toc286401441)

[7. Behavioral Testing Procedure (control participant C1 and C2) 6](#__RefHeading___Toc286401442)

[7.1. Prior to fMRI 6](#__RefHeading___Toc286401443)

[7.2. During fMRI 7](#__RefHeading___Toc286401444)

[8. fMRI Design 7](#__RefHeading___Toc286401445)

[8.1. MT+ Localizer (C1 and C2) 7](#__RefHeading___Toc286401446)

[8.2. Order of experiments 7](#__RefHeading___Toc286401447)

[9. fMRI Data Analysis 8](#__RefHeading___Toc286401448)

[9.1. Pre-processing and coregistration 8](#__RefHeading___Toc286401449)

[9.2. Cortical Surface Reconstruction 8](#__RefHeading___Toc286401450)

[9.3. Voxelwise Analysis - Significance Thresholds 8](#__RefHeading___Toc286401451)

[9.4. ROI Analysis - ROI Selection and Significance Thresholds 9](#__RefHeading___Toc286401452)

[10. Supplementary References 11](#__RefHeading___Toc286401453)

## 1. Participants EB and LB – Case Histories

Both participants were right-handed males. EB had partial sight up to age 13 months. Then his eyes were removed due to retinoblastoma. LB had normal sight until age 14 years. At age 14 he lost sight over the course of several months due to optical nerve atrophy. Neither participant reported having any other history of medical or neurological illness. Both participants have college degrees and are highly functional. Both possess a good deal of insight into their echolocation abilities (e.g., they both routinely teach other blind individuals how to echolocate). Cursory evaluation of anatomical MRI scans by a neurologist suggested that EB’s brain appeared for all intents and purposes ‘normal’ in terms of cortical density, but that LB has signs of slight cortical atrophy in the occipital lobe, especially around the calcarine fissure (J. Ween, personal communication). A difference in cortical structure between EB and LB is not unexpected given their differential case histories, i.e. early versus late onset blindness. As of today, however, the exact nature of cortical differences between late and early onset blindness is unclear (e.g. compare results in Jiang et al., 2009 and Lepore et al, 2010). Atrophy around the calcarine fissure in LB is generally consistent with previously reported cortical changes after optical nerve atrophy (Kitajima et al., 1997) and is in keeping with what would be expected when a well-developed visual sensory system becomes suddenly and irrevocably deprived of visual input. Evidently, the results reported here also raise the issue as to how the use of echolocation, blindness and cortical changes are related.

## 2. Binaural Microphone Calibration

Sounds were recorded (16-bit, stereo, 44.1 kHz sampling rate; Edirol R-09 digital wave recorder) using in-ear binaural omni-directional microphones (Sound Professionals-TFB-2; ‘flat’ frequency range 20 – 20,000 Hz ) placed directly at the opening of the auditory canals and held in place by soft rubber ‘horn-shaped’ housings that conformed to the shape of the concha. Offline calibration sessions determined that amplification of the right channel by 0.78 dB SPL provided an average between-channel correspondence of 0.01 dB SPL (±0.29 dB standard deviation). This amplification was subsequently applied to all in-ear audio recordings prior to any sound editing.

## 3. Sound Equalization

Audio stimuli in all passive listening experiments (including those outside the MRI) were delivered using Sensimetric MRI-compatible insert earphones (model S14) which have a reliable, yet non-flat, frequency response up to 10 kHZ (see also fMRI Design - Audio System). Frequency response equalization (0 – 10 kHz) was achieved by pre-filtering the sound files using equalization filters provided by the headphone manufacturer (Sensimetrics, Malden, MA, USA).

## 4. Description of Echolocation Clicks

The echolocation sounds made by both individuals were orally produced vacuum pulses, i.e. clicks. EB used palatal clicks, whereas LB used alveolar clicks. Regardless of participant and context, each click was approximately 10 ms in duration and had a broad frequency spectrum (see Figure 1A). In our experiment, EB and LB produced clicks at a stimulus onset asynchrony of at least 500 ms, ensuring that there was no temporal overlap between echoes from previous clicks and the onset of the next click. Both EB and LB mentioned, however, that they typically adapt the timing of clicks to the demands of the situation. For example, during bike riding clicks will be made at a more frequent rate than during slow walking. In the anechoic chamber, amplitude measurements (Larson-Davis System 824) of typical echolocation clicks measured with the amplitude meter located at the ear (EB) varied between 71 and 74 dB SPL. At distances of 1m and 2m from the echolocator, peak amplitudes of approximately 53.6 and 49.0 dB SPL, respectively, were registered. When asked to produce a ‘loud’ click, peak intensities of 63 and 62 dB SPL were registered at 1m and 2m, respectively.

## 5. Experimental Stimuli - Spatial Source Localization

### 5.1. Setup and General Recording Procedure (Anechoic Chamber)

*Recording Procedure ‘Empty Clicks’:*

For each participant we made recordings while no object was present, i.e. ‘empty’ click recordings, which were later used to create sounds for spatial source localization. Each participant (either EB and LB) produced at least 20 empty echolocation clicks with his head held stationary and straight ahead. Echolocation clicks occurred at stimulus onset asynchronies (SOA) of at least 500 ms.

*Recording Procedure Spatial Source Localization*

To create stimuli for spatial source localization modified empty clicks from EB and LB (see Experimental Stimuli and Sound Editing below) were played through computer speakers (Hewlett Packard Mini USB-Powered Laptop Speakers, Model NN109AA#ABA). The speakers were mounted vertically on top of one another on top of a 0.5 cm diameter telescopic steel pole at a height of 1.3 m. The pole was turned so that the speakers faced the listener, who held his head stationary straight ahead. The pole was placed at a radial distance of 150 cm at various angular positions around the listener (i.e. straight ahead and 36°, 27°, 18°, 16°, 14°, 12°, 10°, 8°, 6°, 4°, 2°, 1° to the left and right of the straight ahead). Three sample recordings were made for each position of the pole and participant, because we had three modified clicks from each EB and LB, respectively. Due to time constraints, recordings for both participants were made in the ears of one of the experimenters (SA), instead of the ears of EB and LB themselves.

### 5.2. Experimental Stimuli and Sound Editing

To create stimuli for spatial source localization, we first extracted 3 unique empty click sequences (each containing approximately 5 - 6 empty clicks) from the empty click recordings from each participant. Subsequently, the stereo sound was transformed into a mono sound by randomly choosing either the left or right channel track and replicating it in the other channel. Finally, the mono-track waveform samples were inverted and reversed. To record spatial source sounds, the sounds created this way were played through computer speakers at the 25 different source locations (i.e. 36°, 27°, 18°, 16°, 14°, 12°, 10°, 8°, 6°, 4°, 2°, 1° to the left and right of the straight ahead) and recorded in SA’s ears. From these recorded source sounds 3 trains of 5 s click sequences (approx. 5 – 6 clicks) were extracted to create 3 unique sample sounds for each of the 25 external sound locations for each participant, thus creating 75 sound stimuli for each participant.

## 6. Behavioral Testing Procedures (EB and LB)

### 6.1. Prior to fMRI

*Angular Position Discrimination - Active Echolocation*

To determine angular position discrimination thresholds we employed a 2-Interval-2-Alternative-Forced-Choice adaptive staircase method. The participant’s task on every trial was to determine whether a position marker (described in main text) at a test position was located to the left or right of a position marker at a straight ahead reference position. Presentation was sequentially, such that the marker was always presented first at the straight ahead reference position and then at the test position. The marker was always presented at a radial distance of 150 cm.

To minimize the possibility of procedural bias, two intertwined staircases were used that approached the reference position (0°) from the left or right (-36° or 36° starting value, respectively). Presentation order of staircases was pseudo-random such that one staircase would not run for more than 4 consecutive trials. The angular difference between test and reference on each trial was determined adaptively. In the first two trials we used the stochastic approximation by Robbins-Monro (Robbins and Monro 1951):

$$x_{n+1}=x_{n}-\frac{c}{n(z_{n}-\phi)}$$

where n is the number of the current trial, X the value of the stimulus, and c the initial step size (set at 36°), $\phi$is the probability of responding in a correct or incorrect way with respect to the corresponding staircase (0.5 in our paradigm) and Z defines if the response was correct (1) or incorrect (0), referring to the corresponding staircase (e.g., ‘left’ is correct for the left- and incorrect for the right-starting staircase). For subsequent trials we used the accelerated stochastic approximation by Kesten (1958):

$$x_{n+1}=x_{n}-\frac{c}{(2+m)(z_{n}-\phi)}$$

which additionally includes *m* for the number of changes in the response category, i.e., *m* increased by one when the response switched from left to right, or vice versa, in one staircase. The test was terminated when the participant’s responses appeared to have converged sufficiently to permit estimation of thresholds. One session took approximately 1 hour to complete.

At the beginning of each trial, the participant would place earmuffs over his ears. Then, the experimenters would place the position marker at the straight ahead reference position. Then, the experimenters retracted to the back of the anechoic chamber, behind the participant. Once the experimenters were behind the participant, one of them would slightly tap the shoulder of the participant. On that signal the participant removed the earmuffs and echolocated until he had a good sense of where the marker was located. Then, the participant would replace the earmuffs over the ears. Once the ears were covered, one of the experimenters would place the pole at the test position, while the other experimenter would walk around such as to provide additional sound masking in the event that the earmuffs did not eliminate all acoustic energy. After the pole had been placed, both experimenters would again retract to the back of the room and signal the participant with a shoulder tap. The participant would then remove the earmuffs and echolocate until he had a good sense of where the marker was located. Then, the participant would state whether the test was located to the right or left of the reference. The experimenter would enter the response into a computer keyboard. The computer would then calculate the test position for the next trial.

Before the experiment started, the experimenter explained the task and procedure to the participant. In addition, the participant was told that it might become increasingly more difficult to determine the position of the test marker with respect to the reference, and that this was a consequence of the procedure used. The participant was told that if he was uncertain about the position of the test with respect to the reference, he should respond with his “best guess”. The participant was also asked to keep his head stationary straight ahead during presentation of the markers and in between presentation of the reference and the test. EB and LB participated in separate sessions.

*Angular Position Discrimination – Passive Listening*

During passive listening we used exactly the same 2-interval-2-alternative forced choice procedure as during active echolocation. The main difference to the active echolocation task was that participants did not actively echolocate, but that they listened to recordings of their own clicks and echoes using the MRI compatible headphones. As described above, recordings had been obtained for each participant separately and for various positions of the position marker. Participants listened sequentially to sound samples, such that they listened to the reference first and the test second. After listening to the test the participant indicated whether the recording that he had heard during test had been made with a position marker placed to the left or right, as compared to the recording that they had heard during the reference. Participants could listen to each sample as often as they wished. Just as in the active echolocation task, the experimenter keyed the participant’s response into the computer and the computer selected the test stimulus for the next trial using an adaptive procedure. The experiment was conducted in a quite hotel room, while the participant was seated and held his head stationary.

For practical reasons, stimulus recordings had been made for only a subset of locations of the position marker. However, test values for threshold determination were computed on a continuous scale (see equations above). The testing procedure was therefore adapted as follows: On each trial, the requested test value was computed by the computer. If the requested recording was available, that recording was played. However, if the requested recording was not available, a recording was chosen that had been made with a position marker at a location closest to the location used in the requested recording. This ‘nominal’ test recording was then presented. The requested test value for the next trial was then computed based on the nominal test value and the participant’s response. Plotted in Fig.1 are the test values obtained this way.

*Angular Position Discrimination – Source Localization*

During angular position discrimination via source localization, the exact same procedure as during passive listening was used. The only difference was that instead of listening to echolocation recordings participants now listened to recordings of source sounds.

### 6.2. During fMRI (see main text: fMRI functional and behavioral paradigm)

### 6.3. After fMRI

Due to a programming error, behavioral responses for shape/location and motion experiments during fMRI had not been saved to disk. Thus, an additional behavioral testing session was conducted. The behavioral test was conducted at the end of the second day of fMRI testing in a quite hotel room. Upon questioning both participants reported that the difficulty (or ease) of the task felt equivalent to the difficulty (or ease) of the task when it had been performed in the fMRI scanner.

*Shape/Location*

Stimuli were played using the same timing and instructional cues as in the fMRI. Since the experiment was conducted outside of the scanner, no intermittent scanner noise was present in between stimulus presentations. Furthermore, the participant did not press a button, but gave a spoken response (i.e. left, right, flat or concave), which was recorded by the experimenter. To preserve statistical power, while minimizing the burden from the perspective of the participants (after all it had been a very long week of testing) the number of trials was reduced from 80 (used in fMRI) to 64 (32 for shape and 32 for location conditions).

*Motion*

The procedure was the same as for the Shape/Location experiment. Numbers of trials was reduced from 80 (used in fMRI) to 64.

## 7. Behavioral Testing Procedure (control participant C1 and C2)

### 7.1. Prior to fMRI

Control participant C1 and C2 listened to sound stimuli recorded and used with EB and LB, respectively, during shape, location, motion and scenes experiments. Both control participants were first made familiar with the sounds and then performed practice sessions for each experiment (practice time was slightly more than 1 hour for each participant). The practice session mimicked the behavioral paradigm during fMRI scanning, but during practice both control participants received feedback from the experimenter regarding the “identity” of the sound and the correctness of their classification response. Responses for practice sessions were not recorded.

### 7.2. During fMRI

During fMRI, control participant C1 and C2 performed exactly the same tasks as EB and LB, respectively. In-scanner response acquisition worked perfectly in all experiments for C2. For C1, several responses were lost because button presses did not register reliably. However, the number of responses obtained for C1 was still large enough to have sufficient statistical power.

## 8. fMRI Design

### 8.1. MT+ Localizer (C1 and C2)

During MT+ localizer runs participants viewed displays through a front-surface mirror mounted on top of the head coil. The display was projected with an LCD projector (AVOTEC Silent Vision Model 6011, Avotec, FL, USA) on a rear-projection screen located behind the head-coil in the bore. The display showed a circular red fixation target (0.5° visual angle diameter) in three different conditions: *baseline* (in front of a black background), *stationary*: (in front of a pattern of white random dots, diameter 0.1° visual angle, spaced on average 1 degree of visual angle apart, that flickered at 1 Hz) and *moving* (in front of pattern of white random dots, diameter 0.1° visual angle, spaced on average 1 degree of visual angle apart, that translated smoothly across the visual field and changed direction of motion randomly every second). The active display area subtended 26° x 26° of visual angle. Each condition was shown in blocks of 16 seconds and in counterbalanced order, such that baseline always preceded any two motion and stationary blocks and that moving and stationary blocks occurred in alternating order (i.e. stationary-moving followed moving-stationary and vice versa). Each run began and ended with a baseline block. The total number of blocks in each run was 25 (8 moving, 8 stationary and 9 baseline). Prior to the start of functional scanning, a shimming procedure was employed to reach magnetic field homogeneity. Hence, the duration of each run was 25 x 16 s and the number of functional volumes acquired in each run was 200. Each participant performed 2 runs. Participants task was to direct their gaze at the fixation target in all conditions.

Due to a projector malfunction during MT+ localizer runs with C1 (i.e. C1 reported that the display was barely visible), we used MT+ localizer data that had been acquired in a different session using a slightly different localizer stimulus (but exactly the same imaging parameters and setup). In that session C1 had viewed displays that showed the same circular red fixation target in stationary and moving conditions in alternating order. Each run began and ended with a stationary block. The total number of blocks in each run was 17 (8 moving, 9 stationary). Prior to the start of functional scanning, a shimming procedure was employed to reach magnetic field homogeneity. Hence, the duration of each run was 17 x 16 s and the number of functional volumes acquired in each run was 136. C1 had performed 2 runs.

### 8.2. Order of experiments

All participants performed the experiments in the following order:

1: shape/location, 2: motion, 3: scenes, 4: MT+-localizer (C1 and C2 only). All participants (except C2 who ran all experiments in one day) ran shape/location and motion experiments on one day and the scenes experiment on the next. Two anatomical scans were taken for each EB and C1, one at the beginning of each scanning session. Three anatomical scans were taken for LB and C2, one at the beginning of each experiment.

## 9. fMRI Data Analysis

### 9.1. Pre-processing and coregistration

In the very beginning of each run a dummy volume had been acquired. For echolocation runs, this dummy volume was discarded from functional data analysis, but used for functional-to-anatomical coregistration. For localizer runs, all volumes were used for functional analysis. Each run was subjected to Slice Scan Time Correction (Tri-linear sinc), temporal High-Pass Filtering (cutoff at 2 sines/cosines) and 3D Motion correction (Tri-linear sinc). For quality assessment we inspected the motion parameters estimated for within-run motion for each run. This inspection revealed that motion estimates did not exceed 1 degree rotation and 1 mm translation within any given run. Furthermore, motion parameter estimates appeared drift-like and uncorrelated with the paradigm. To align the functional data for each participant across runs within a session, we first used 3D motion correction to align each volume within a run to the functional volume closest to the anatomical scan from the same session. To align functional data across sessions for each participant, we then coregistered the anatomical from each session to a general anatomical target of that same participant. The general anatomical target was chosen deliberately for each participant. The general anatomical target for each participant was then transformed into standard stereotactic space (Talairach and Tournoux, 1988).

### 9.2. Cortical Surface Reconstruction

Cortical Surface Reconstruction for each participant was performed in standard stereotactic space. An initial gray-white matter boundary was obtained using an automatic segmentation algorithm. Where necessary, alterations were performed manually to increase anatomical accuracy. The gray-white matter boundary obtained this way was then transformed into a 3D-surface mesh, which was subsequently smoothed (smoothing force .03; 300 iterations) and flattened (smoothing force .8; 500 iterations; surface area kept constant). Functional activity was projected onto the flattened mesh by sampling the statistical result in volume space along the surface normals of the folded mesh (read depth -1 to 3mm) and projecting the average of this sample onto the flattened cortical surface. Volumetric statistical data was interpolated to the resolution of the anatomical data (1x1x1 mm) before sampling.

### 9.3. Voxelwise Analysis - Significance Thresholds

*BOLD activity related to echolocation as compared to silence (Fig.2, 3, top panels)*

The significance threshold for evaluation of results in volume space was set to 0.1 (Bonferroni corrected), where the correction took into account all voxels in the functional volume. This threshold was selected because it removed obvious false positives such as activations outside of the brain, while showing positive activation in expected areas (i.e. in auditory cortex). To increase power, especially for our control participants, we also tried more liberal voxel-wise statistical thresholds (i.e. p<.001 or p<.0005 or p<.0001) in combination with cluster size thresholds. However, when we applied cluster size thresholds as determined with the minimum cluster size threshold estimator plugin provided in brain voyager (Forman et al., 1995; Goebel et al., 2006), activity outside of the brain remained. Thus, this method could not distinguish obvious false positives (outside of the brain) from potentially real activity in our data, which is why we did not use it. As it turned out, a .1 (Bonferroni corrected) threshold in volume space corresponded very closely to a .05 (Bonferroni corrected) threshold in surface space for each participant. Hence, we applied a .05 threshold (Bonferroni corrected) to the cortical data in surface space and a threshold of .1 (Bonferroni corrected) to the cerebellum data in volume space.

*BOLD activity related to outdoor sounds as compared to silence (Fig.4, 5, top panels)*

Thresholds were chosen as for ‘echolocation sounds compared to silence’

### 9.4. ROI Analysis - ROI Selection and Significance Thresholds

*Left and right calcarine (EB and LB only)*

As described in the main text, to define regions of interest in calcarine cortex for EB and LB we selected voxels along the anatomically defined left and right calcarine for which the contrast (Echo_Moving_ + Echo_Stationary_)> 0 was significant. Sometimes activity filled the volume space between the left and right hemispheres (especially for EB who had a lot of activity in his right calcarine cortex). Thus, to avoid ‘bleeding in of activity’ from the right to the left and vice versa, we defined a 6 mm voxel selection gap between left and right calcarine. With our fMRI resolution, a gap of 6 mm corresponds roughly to the space sampled by two functional voxels. The level of significance was set to p<.001 for the left calcarine in EB and the left and right calcarine in LB. In addition, we imposed a cluster size threshold of 10. The right calcarine of EB was overrun with activity and it was virtually impossible to detect clusters boundaries with a threshold p<.001. Hence, to avoid selecting the whole right calcarine and to make cluster sizes somewhat more similar across the left and right hemispheres, the threshold for the right calcarine in EB was increased. The ROI results reported in the main text were computed based on a cluster selected with t(118)=8.5, p = 6.8^-14^. However, we confirmed with additional statistical analysis that the results of our ROI analysis did not depend on the threshold of cluster selection.

*Left and right Heschl’s gyrus (EB and LB only)*

As described in the main text, to define regions of interest in Heschl’s gyrus for EB and LB we selected voxels along the anatomically defined left and right Heschl’s gyrus (which contains the PAC) for which the contrast (Echo_Moving_ + Echo_Stationary_)> 0 was significant. For all participants and hemispheres the level of significance was set to p<.2.2^-8^, which corresponds to t(118)=6. In addition, we imposed a minimum cluster size threshold of 108 voxels.

*MT+ (C1 and C2 only)*

*C1:* To obtain activity related to processing of visual motion we applied a GLM with one predictor “moving” to the z-transformed time courses of runs obtained in localizer experiment (2 runs total). Predictors were obtained by convolving a boxcar function that indicated the presence of the event with the standard 2-gamme HRF. The GLM was run as a fixed effect model. MT+ was then defined by selecting voxels posterior to the ITS/LOS junction for which the beta value was significantly larger than zero. MT+ was selected using both a liberal voxelwise p<.05 threshold and more conservative Bonferroni corrected p<.05 threshold, where the correction was computed based on all voxels in the functional volume. Table S10 shows center of gravity Talairach coordinates for MT+ ROIs.

*C2:* To obtain activity related to processing of visual we applied a GLM with two predictors, i.e. “moving” and “stationary” to the z-transformed time courses of runs obtained in localizer experiment (2 runs total). Predictors were obtained by convolving a boxcar function that indicated the presence of the event with the standard 2-gamme HRF. The GLM was run as a fixed effect model. To determine BOLD activity related to the processing of visual motion, we used the contrast (moving>stationary). MT+ was then defined by selecting voxels posterior to the ITS/LOS junction for which the value of the contrast was significant. MT+ was selected using both a liberal voxelwise p<.05 threshold, and more conservative Bonferroni corrected p<.05 threshold, where the correction was computed based on all voxels in the functional volume. Table S10 shows center of gravity Talairach coordinates for MT+ ROIs.

## 10. Supplementary References

Blauert J (1998) Spatial Hearing: The Psychophysics of Human sound localization (rev. ed). Massachusetts: MIT Press.

Forman SD, Cohen JD, Fitzgerald M, Eddy WF, Mintun MA et al. (1995) Improved assessment of significant activation in functional magnetic resonance imaging (fMRI): use of a cluster-size threshold. Magnetic Resonance in Medicine 33: 636–647.

Goebel R, Esposito F, Formisano E (2006) Analysis of functional image analysis contest (FIAC) data with brainvoyager QX: From single-subject to cortically aligned group general linear model analysis and self-organizing group independent component analysis. Human Brain Mapping 27(5): 392-401.

Kitajima M, Korogi Y, Hirai T, Hamatake S, Ikushima I, et al. (1997) MR changes in the calcarine area resulting from retinal degeneration. Am J Neuroradiol 18:1291–1295.
